# Surface and Extracellular Proteome of the Emerging Pathogen *Corynebacterium ulcerans*

**DOI:** 10.3390/proteomes6020018

**Published:** 2018-04-17

**Authors:** Miriam Bittel, Susanne Gastiger, Bushra Amin, Jörg Hofmann, Andreas Burkovski

**Affiliations:** 1 Professur für Mikrobiologie, Friedrich-Alexander-Universität Erlangen-Nürnberg, Staudtstr. 5, 91058 Erlangen, Germany; miriam.bittel@uk-erlangen.de (M.B.); susanne.gastiger@fau.de (S.G.); 2 Lehrstuhl für Biochemie, Friedrich-Alexander-Universität Erlangen-Nürnberg, Staudtstr. 5, 91058 Erlangen, Germany; bua4@pitt.edu (B.A.); joerg.hofmann@fau.de (J.H.); 3 Department of Chemistry, University of Pittsburgh, Pittsburgh, PA 15260, USA

**Keywords:** diphtheria, exoproteome, genomics, host-pathogen interaction, surface proteome, virulence factors

## Abstract

*Corynebacterium ulcerans* is an emerging pathogen, which is increasingly recognized as an etiological agent of diphtheria, but can also evoke ulcers of the skin and systemic infections in humans. Besides man, the bacteria can colonize a wide variety of different animals, including cattle and pet animals, which might serve as a reservoir for human infections. In this study, surface-located proteins and the exoproteome of two *Corynebacterium ulcerans* strains were analyzed, since these may have key roles in the interaction of the pathogen with host cells. Strain 809 was isolated from a fatal case of human respiratory tract infection, while strain BR-AD22 was isolated from a nasal swap of an asymptomatic dog. While a very similar pattern of virulence factors was observed in the culture supernatant and surface protein fractions of the two strains, proteome analyses revealed a higher stability of 809 cells compared to strain BR-AD22. During exponential growth, 17% of encoded proteins of strain 809 were detectable in the medium, while 38% of the predicted proteins encoded by the BR-AD22 chromosome were found. Furthermore, the data indicate differential expression of phospholipase D and a cell wall-associated hydrolase, since these were only detected in strain BR-AD22.

## 1. Introduction

*Corynebacterium ulcerans* is a pathogenic member of the genus *Corynebacterium*, which is part of the family *Corynebacteriaceae*, the order *Actinomycetales*, and the phylum *Actinobacteria* [1]. *C. ulcerans* was first described by Gilbert and Stewart, who isolated the bacteria from the throat of a patient with respiratory diphtheria-like illness [2]. In fact, when lysogenized by a *tox* gene-carrying corynephage, *C. ulcerans* can—similar to *Corynebacterium diphtheria*—produce diphtheria toxin and evoke the typical symptoms of respiratory diphtheria (for a recent review on corynophages, see Reference [3]).

*C. ulcerans* is an emerging pathogen [4,5,6,7,8], and during the past decade diphtheria-like infections with toxigenic *C. ulcerans* have outnumbered those caused by toxigenic *C. diphtheriae* in many industrialized countries [9]. Moreover, during the last years, other human infections associated with *C. ulcerans* appear to be increasing in various countries, and can most often be ascribed to zoonotic transmission (for an example, see References [10,11]; for a review, see Reference [7]). The range of mammals that may serve as a reservoir for human infections is extremely broad. *C. ulcerans* was isolated from cattle, goats, pigs, wild boars, dogs, cats, ground squirrels, otters, camels, monkeys, orcas, and water rats (for a review, see Reference [7]).

In 2011, two *C. ulcerans* strains from the metropolitan area of Rio de Janeiro, Brazil, were sequenced [12]—BR-AD22, isolated from a nasal swab of an asymptomatic dog, and 809, isolated from a bronchoalveolar lavage sample of an 80-year-old woman with fatal pulmonary infection [13,14]. Based on these genome sequences and comparative genomics approaches, a number of putative virulence factors were annotated. Unfortunately, functional analyses are scarce, and up to now, a limited number of data concerning adhesion and invasion of epithelial cells, interaction with macrophages, fibrinogen, fibronectin and collagen binding, antimicrobial profiles, and arthritogenic potential of isolates were published [15,16,17,18]. To get deeper insights into *C. ulcerans* physiology and pathology, proteome analyses were carried out. Similar studies focusing on different corynebacteria, e.g., *C. diphtheriae*, *Corynebacterium jeikeium*, and *Corynebacterium pseudotuberculosis* strains have been previously published [19,20,21,22,23,24,25,26]. The study presented here describes proteome analyses of *C. ulcerans* strains 809 and BR-AD22, focusing on surface-located and medium-secreted proteins of the two strains, since these proteins may represent key components of pathogen-host interaction, and may influence pathogenicity as well as immune response.

## 2. Materials and Methods

### 2.1. Strains and Growth Conditions

*C. ulcerans* 809 and BR-AD22 were grown in heart infusion (HI) medium (25 g HI broth; Becton, Dickinson, ND, USA). For solid media, 15 g/L Bacto Agar (Oxoid, Basinstoke, UK) was added. Incubation of liquid cultures of *C. ulcerans* was carried out at 30 °C under shaking in baffled flasks. Overnight cultures were used to inoculate fresh media to an OD_600_ of approximately 0.1, bacteria were incubated at 30 °C until the exponential growth phase was reached (OD_600_ approximately 2), and cultures were further processed as described below.

### 2.2. Isolation of Extracellular Proteins Secreted into the Medium

For preparation of extracellular proteins, the cells were separated by centrifugation (25 min, 5000× *g*, 4 °C) and Complete EDTA-free protease inhibitor cocktail (Roche, Mannheim, Germany) was added to the supernatant to avoid proteolysis. To prevent contamination of extracellular proteins by cells or cell debris, the supernatant was centrifuged again (1 h, 7000× *g*, 4 °C) and subsequently filtered using 0.2 µm pore-size filters (Minisart, Sartorius, Göttingen, Germany) [27]. Proteins were precipitated under constant stirring by dropwise addition of trichloroacetic acid (10% *w*/*v*) and incubation at 4 °C overnight. Precipitated proteins were harvested by centrifugation (1 h, 5500× *g*, 4 °C). Pellets were washed three times with acetone, incubated for 5 min on ice and centrifuged (10 min, 5500× *g*, 4 °C). This step was repeated twice with 80% acetone and once with 100% acetone. The proteins were air-dried under sterile conditions and resuspended in 250 µL dehydration buffer (8 M urea, 50 mM Tris, 20 mM DTT, 1% sodium desoxycholate).

### 2.3. In-Solution Tryptic Digest

A total of 1.5 µg of proteins (see Section 2.2) were transferred to 10 kDa Hydrosart^®^ membrane filters (Sartorius Stedim biotech, Göttingen, Germany) for modified filter-aided sample preparation (FASP) [28]. Proteins were reduced with 250 µL of reduction buffer containing 8 M urea, 100 mM triethylammonium bicarbonate buffer (TEAB, Sigma-Aldrich, Munich, Germany), and 20 mM dithiothreitol (DTT) for 30 min at room temperature. Alkylation of sulfhydryl groups was carried out in the same buffer containing 40 mM chloroacetamide, instead of DTT for 30 min. Proteins were washed with 250 µL of 1 M urea in 50 mM TEAB and trypsinized with 1 µg of sequencing-grade trypsin (Promega, Mannheim, Germany) in 1 M urea in 50 mM TEAB for 18 h at 37 °C. Peptides were extracted by centrifugation and desalted on C18 stage tips. Prior to nanoLC-MS/MS analysis, tryptic peptides were dried under vacuum and resuspended in 0.1% trifluoroacetic acid (TFA).

### 2.4. NanoLC-MS/MS Analysis

Mass spectrometric analyses by nanoLC-MS/MS were carried out as described [27]. In short, resulting peptides (approx. 1 µg) were loaded on a nanoflow Ultimate 3000 HPLC (Dionex, Sunnyvale, CA, USA) for separation on EASY-Spray column (Thermo Fisher Scientific; C18 with 2 μm particle size, 50 cm × 75 μm), with a flow rate of 200 nL/min by increasing acetonitrile concentrations over 120 min. All samples were analyzed on an Orbitrap Fusion (Thermo Fisher Scientific, Waltham, MA, USA) with the previously described MS/MS settings [27]. The mass spectrometer was operating with 2000 V spray voltage, 300–2000 (*m*/*z*) scan range, a maximum injection time of 50 ms, and an AGC target of 400,000 for the first stage of mass analysis t (MS^1^). The most intense ions were selected for collision-induced dissociation with collision energy of 35%, a maximum injection time of 250 ms, and an AGC target of 100 for the second stage of mass analysis (MS^2^).

Raw data files were processed against *C. ulcerans* databases 809 (UniProt UP 000008886, rel.1/16, 2180 sequences) and BR-AD22 (UniProt UP000008887, rel. 2/16, 2335 sequences), using Proteome Discoverer 2.0 (Thermo Scientific, Waltham, MA, USA). Theoretical masses were generated by trypsin with a maximum of two missed cleavages for full-tryptic and semi-tryptic peptides, and their product ions were compared to the measured spectra with the following parameters: carbamidomethyl modification was set as fixed and oxidation of methionine residues and carbamidomethylated lysine residues were set as an optional modification. Mass tolerance was set to 10 ppm for survey scans and 0.5 Da for fragment mass measurements. Peptide charges of 2–7 were allowed. Only resulting peptides with false discovery rate (FDR) below 1% were regarded as identified.

### 2.5. Isolation of Cell Surface Proteins by Tryptic Shaving

The preparation of surface proteins was based on a previously published protocol [29]. Harvested cells were resuspended in PBS and washed three times in this buffer (137 mM NaCl, 2.7 mM KCl, 10 mM Na_2_HPO_4_, 2 mM KH_2_PO_4_, pH (HCl) 7.4). Cells were treated with 25 U sequencing-grade trypsin (Promega, Mannheim, Germany) in 500 µL PBS buffer, for 15 min at 37 °C. Subsequently, bacteria were separated by centrifugation (30 min, 4 °C, 13,000× *g*), and the supernatant was filtered using 0.2 µm pore-size filters (Minisart, Sartorius, Göttingen, Germany). Mass spectrometry was carried out as described.

### 2.6. Zymography

To test for protease activity, the protein fractions were analyzed using casein gels for zymography [30]. The samples were prepared in a non-reducing loading buffer (125 mM Tris, 20% glycerol, 6% SDS, 0.02% bromophenol blue) and incubated for 25 min at room temperature. A 10% polyacrylamide gel containing 0.1% casein was used for electrophoresis. After electrophoresis, SDS was removed by incubation for 30 min in 2.5% Triton X-100. The gel was equilibrated for 30 min and subsequently incubated for 12 h at 37 °C in digestion buffer (50 mM Tris, 200 mM NaCl, 5 mM CaCl_2_ × 2 H_2_O, 240 mM Brij35 (pH 7.4)). Staining was carried out by incubation for 5 h in 40% methanol, 7% acetic acid, 0.5% Coomassie brilliant Blue R. Gels were destained by 15 min incubation in 40% methanol, 7% acetic acid.

### 2.7. Inverse CAMP Test

For detection of phospholipase D activity, an inverse CAMP (Christie–Atkins–Munch-Petersen) test was carried out [31]. For this purpose, *Staphylococcus aureus* ATCC 29213 was streaked-out as a vertical line and *C. ulcerans* BR-AD22 and 809 as parallel horizontal lines on Columbia Blood Agar plates (Oxoid, Basingstoke, UK), and bacteria were incubated for two days at 37 °C.

## 3. Results

### 3.1. Analysis of Proteins in Extracellular and Surface Fraction

When supernatants of *C. ulcerans* strains 809 and BR-AD22 cultures grown to exponential phase were analyzed, strain-dependent differences in the number of proteins were observed. In strain 809, 221 proteins were identified in three biological replicates, and an additional 144 proteins in less than three replicates, leading to a total number of 365 identified proteins. In contrast, 517 proteins were identified in three biological replicates, and an additional 369 in less than three replicates, leading to a total number of 886 proteins for strain BR-AD22. 134 proteins were identified in culture supernatants of both strains (for supporting information, see Tables S1 and S2). The high number of proteins observed in BR-AD22 supernatants, 38% of the predicted proteins encoded by its chromosomal DNA [12], indicated a considerable lysis of bacterial cells occurring under standard growth conditions in shaking flasks. In contrast, strain 809 was less prone to cell damage. In this case, only 17% of the predicted proteins [12] were detected.

Surface-located proteins were released by trypsin treatment of cells. When corresponding fractions of *C. ulcerans* 809 and BR-AD22 were analyzed after trypsin-shaving, isolation of the supernatant, and subsequent mass spectrometry, again, fractions of strain 809 revealed less proteins compared to BR-AD22. For 809, a total 528 proteins was identified in the shaving fraction, with 305 protein present in triplicates and 223 in less than three samples. For strain BR-AD22, 1025 proteins were identified, with 577 proteins found in triplicates and 448 in less than three samples.

**Figure 1:**
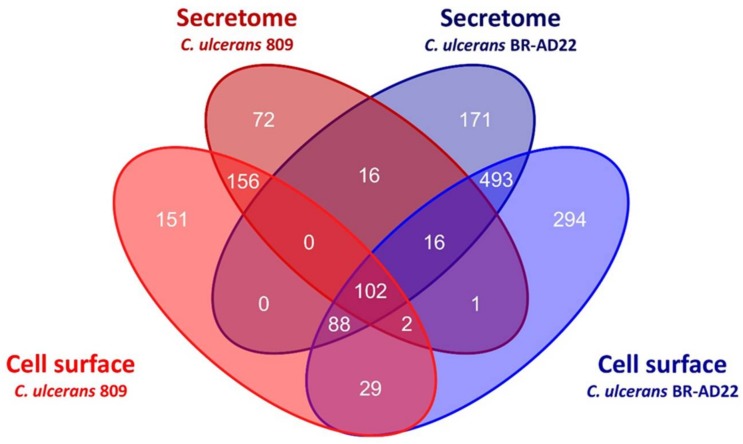
Overview of proteomic data from *C. ulcerans* 809 and BR-AD22. Venn diagram mapping the overlapping proteins in culture supernatant and cell surface fraction.

From the 102 proteins found in all samples, 23 were attributed to ribosome function, 13 were annotated as uncharacterized proteins, and 15 as putative secreted proteins. Only two were annotated in the databases as extracellular (Figure 1, Table 1). In summary, the annotation of the two *C. ulcerans* genomes used is rather preliminary today, and comprises a high number of hypothetical and uncharacterized proteins. Nevertheless, the data obtained here show that proteome analysis may be helpful for a basic characterization of protein expression and localization.

### 3.2. Identification of Putative Virulence Factors

A number of putative virulence factors was annotated in a comparative genome sequencing study of *C. ulcerans* 809 and BR-AD22 [12]. When the results of mass spectrometric fingerprint analysis were monitored for these proteins, almost all virulence factors annotated in the genome sequencing project were observed (Table 2). From the annotated virulence factors, only the pilus subunits comprised LPxTG surface anchoring motifs.

**Figure 2:**
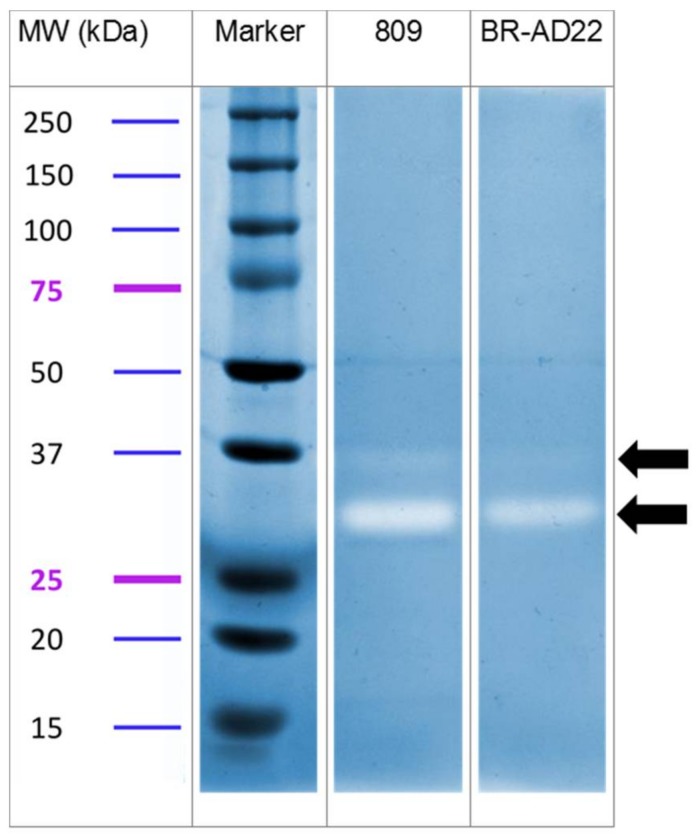
Casein zymography of *C. ulcerans* culture supernatants. Samples were separated using non-reducing, casein-containing gels. After renaturation and incubation in reaction buffer, the gels were stained with Coomassie brilliant blue. Protease activity results in clear bands, which are indicated by arrows. Apparent molecular weights of marker proteins (Dual Colour, Biorad, Munich, Germany) are indicated.

From the 14 annotated virulence proteins, only three were absent in the surface protein fraction and supernatant, namely, a putative ribosome binding protein with homology to Shiga-like toxin, the surface-anchored pilus protein SpaE, and the venom serine protease 2A. In contrast, three other secreted proteases were identified in this study. In fact, protease activity was clearly detectable in samples of supernatants using casein-containing gels. In each fraction, a minor band with an apparent molecular weight of 36 kDa, and a major band with weight 30 kDa, were observed (Figure 2), which fits with the calculated mass of the secreted proteases found. This result indicates a role of proteolysis in pathogenicity of *C. ulcerans*, and, in fact, tissue destruction is often observed in *C. ulcerans* infections.

**Figure 3:**
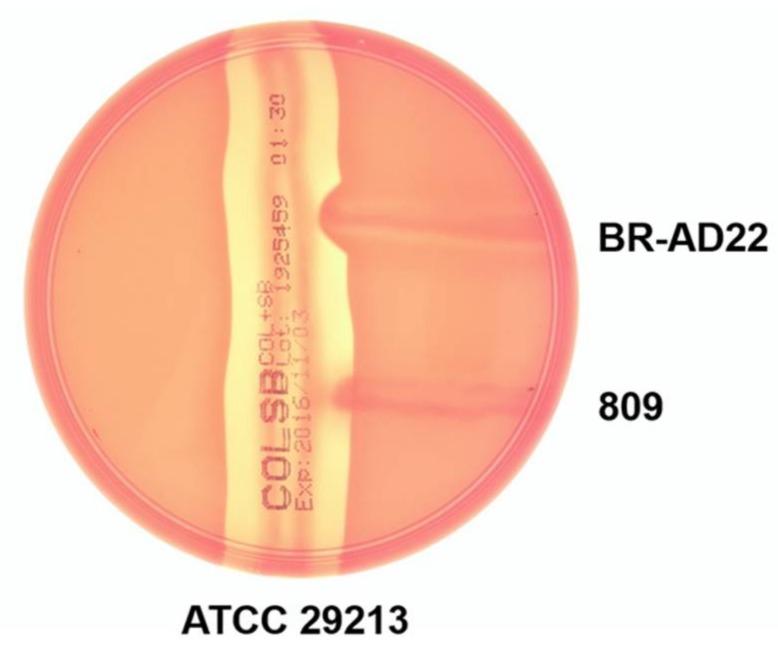
Reverse CAMP test. Hemolysis by *S. aureus* strain ATCC 29213, which is indicated by a halo around the vertical streak-out, is inhibited by strain BR-AD22, while strain 809 had no negative effect on hemolysis.

The results obtained also provide insight into the possible regulation of virulence factors. Although encoded in the genome of the two strains, phospholipase D (PLD) was only found in supernatants of BR-AD22. To verify this result, an inverse CAMP test was carried out. In this assay, hemolysis of sheep erythrocytes by *S. aureus* strain ATCC 29213 is inhibited when phospholipase activity alters the surface of the erythrocytes. In fact, only strain BR-AD22 influences the CAMP reaction of *S. aureus*, supporting expression of PLD in BR-AD22, and its absence in strain 809 (Figure 3).

## 4. Discussion

Proteome analyses of *C. ulcerans* strains grown in vitro indicated that the human isolate 809 is more stable and stress-resistant than strain BR-AD22, which was isolated from an asymptomatic dog. The fact that a considerably high number of intracellular proteins is found at all is rather astonishing, since corynebacteria are typically very robust, due to their complex cell wall structure [32,33,34]. Furthermore, significantly less proteins were found in the supernatant of other pathogenic corynebacteria in previous studies [19,20,21,22,23,24,25,26].

Besides a number of proteins with annotated function, many functionally non-annotated and hypothetical proteins were detected, supporting the idea that proteome analyses may be helpful for a basic characterization of protein expression and localization, even in the case of a very basic genome annotation.

The study presented here was initiated in order to achieve an overview of surface-anchored and secreted proteins, which may have key functions in host interaction. While general expression patterns of putative virulence factors were rather similar for the two investigated strains, one of the main virulence factors of *C. ulcerans*, phospholipase D, was not found in strain 809. In this strain, the ribosome binding protein, exclusively found in its genome sequence, and a cell wall-associated hydrolase were not expressed. The lack of these proteins was unexpected, since strain 809 was isolated from a fatal case of human infection and was expected to be quite pathogenic. These observations highlight that the virulence of *C. ulcerans*, and especially the regulation of its pathogenicity determinants, is hardly understood, and more basic research is necessary to study this emerging human pathogen in order to predict the outcome of future infections. Further proteome studies might be helpful in this respect, especially in combination with other -omics analyses of host-pathogen interactions [35].

## Figures and Tables

**Table 1:** List of proteins identified in all fractions of *C. ulcerans* 809 and BR-AD22. Accession numbers, annotated functions, and cellular localization, as indicated in the different data bases, are given.

Accession No.	Description	Cellular Localisation
G0CTA8; embl-cds:AEG81534	50S ribosomal protein L35	ribosome
G0CTV7; embl-cds:AEG81724	ABC-type transport system involved in Fe-S cluster assembly ATP-binding protein	membrane
G0CU06; embl-cds:AEG81772	Elongation factor EF-P	cytoplasm
G0CU14; embl-cds:AEG81780	Putative secreted protein	
G0CU20; embl-cds:AEG80548	Uncharacterized protein	
G0CU44; embl-cds:AEG81799	Putative secreted protein	
G0CU89; embl-cds:AEG81844	DtxR-family transcription regulator	cytoplasm
G0CU95; embl-cds:AEG81850	Alkyl hydroperoxide reductase	cytoplasm
G0CUC6; embl-cds:AEG80578	Uncharacterized protein	
G0CUG4; embl-cds:AEG80613	Uncharacterized protein	
G0CUM1; embl-cds:AEG81896	Polyribonucleotide nucleotidyltransferase	cytoplasm
G0CUR3; embl-cds:AEG81937	Elongation factor Ts	cytoplasm
G0CUW8; embl-cds:AEG80687	Putative secreted protein	
G0CV31; embl-cds:AEG81974	Uncharacterized protein	
G0CV41; embl-cds:AEG81983	Putative secreted protein	
G0CV58; embl-cds:AEG82000	Putative secreted protein	
G0CV74; embl-cds:AEG82016	Putative secreted protein	
G0CV86; embl-cds:AEG80721	Pyridoxal 5′-phosphate synthase subunit PdxS	cytoplasm
G0CVA1; embl-cds:AEG80736	Nucleoid-associated protein CULC22_00193	cytoplasm
G0CVC7; embl-cds:AEG80762	Aspartokinase	cytoplasm
G0CVK8; embl-cds:AEG82067	Branched-chain-amino-acid aminotransferase	cytoplasm
G0CVR2; embl-cds:AEG80808	Cold shock-like protein A	cytoplasm
G0CVS1; embl-cds:AEG80815	Putative secreted protein	
G0CVT0; embl-cds:AEG80823	Dihydrolipoyl dehydrogenase	cytoplasm
G0CVT9; embl-cds:AEG80832	Putative secreted protein	
G0CVU4; embl-cds:AEG80837	Uncharacterized protein	
G0CVU8; embl-cds:AEG80841	2,3-bisphosphoglycerate-dependent phosphoglycerate mutase	cytoplasm
G0CVX3; embl-cds:AEG80866	Thiol-disulfide isomerase/thioredoxin	cytoplasm
G0CW00; embl-cds:AEG82124	Glutamine--fructose-6-phosphate aminotransferase (isomerizing)	cytoplasm
G0CW81; embl-cds:AEG80892	50S ribosomal protein L11	ribosome
G0CW82; embl-cds:AEG80893	50S ribosomal protein L1	ribosome
G0CW87; embl-cds:AEG80898	50S ribosomal protein L7/L12	ribosome
G0CW95; embl-cds:AEG80906	DNA-directed RNA polymerase subunit beta	cytoplasm
G0CWB3; embl-cds:AEG80924	Elongation factor G	cytoplasm
G0CWB4; embl-cds:AEG80925	Elongation factor Tu	cytoplasm
G0CWC3; embl-cds:AEG80934	30S ribosomal protein S10	ribosome
G0CWC4; embl-cds:AEG80935	50S ribosomal protein L3	ribosome
G0CWC5; embl-cds:AEG80936	50S ribosomal protein L4	ribosome
G0CWC6; embl-cds:AEG80937	50S ribosomal protein L23	ribosome
G0CWC7; embl-cds:AEG80938	50S ribosomal protein L2	ribosome
G0CWC9; embl-cds:AEG80940	50S ribosomal protein L22	ribosome
G0CWD0; embl-cds:AEG80941	30S ribosomal protein S3	ribosome
G0CWD1; embl-cds:AEG80942	50S ribosomal protein L16	ribosome
G0CWD8; embl-cds:AEG80949	50S ribosomal protein L14	ribosome
G0CWE7; embl-cds:AEG80958	50S ribosomal protein L18	ribosome
G0CWL5; embl-cds:AEG82198	50S ribosomal protein L27	ribosome
G0CWL6; embl-cds:AEG82199	50S ribosomal protein L21	ribosome
G0CWM9; embl-cds:AEG82211	ATP-dependent Clp protease proteolytic subunit	cytoplasm
G0CWN2; embl-cds:AEG80960	50S ribosomal protein L30	ribosome
G0CWN3; embl-cds:AEG80961	50S ribosomal protein L15	ribosome
G0CWN5; embl-cds:AEG80963	Maltotriose-binding protein	membrane
G0CWQ1; embl-cds:AEG80980	DNA-directed RNA polymerase subunit alpha	cytoplasm
G0CWS7; embl-cds:AEG81005	10 kDa chaperonin	cytoplasm
G0CWU3; embl-cds:AEG81019	Putative secreted protein	
G0CX97; embl-cds:AEG81093	LytR-family transcription regulator	cytoplasm
G0CXC9; embl-cds:AEG81124	Putative secreted protein	
G0CXG7; embl-cds:AEG82307	Succinyl-CoA:Coenzyme A transferase	redox chain
G0CXJ0; embl-cds:AEG82330	Phosphoribosylformylglycinamidine synthase subunit PurS	cytoplasm
G0CXK8; embl-cds:AEG81127	Uncharacterized protein	
G0CXM1; embl-cds:AEG81140	Uncharacterized protein	
G0CXP5; embl-cds:AEG81163	Carbonic anhydrase	cytoplasm
G0CXQ6; embl-cds:AEG81174	Resuscitation-promoting factor	extracellular
G0CXQ9; embl-cds:AEG81178	Putative secreted protein	
G0CXR6; embl-cds:AEG81185	Citrate synthase	cytoplasm
G0CXR9; embl-cds:AEG81188	Superoxide dismutase [Cu-Zn]	cell envelope
G0CY06; embl-cds:AEG82362	Putative secreted protein	
G0CY28; embl-cds:AEG81208	Uncharacterized protein	
G0CY42; embl-cds:AEG81222	Uncharacterized protein	
G0CY56; embl-cds:AEG81236	50S ribosomal protein L28	ribosome
G0CY58; embl-cds:AEG81238	50S ribosomal protein L32	ribosome
G0CY59; embl-cds:AEG81239	Two-component system transcriptional regulatory protein	cytoplasm
G0CYA4; embl-cds:AEG81281	50S ribosomal protein L25	ribosome
G0CYH1; embl-cds:AEG82442	Purine phosphoribosyltransferase	cytoplasm
G0CYH2; embl-cds:AEG82443	Putative membrane protein	membrane
G0CYK0; embl-cds:AEG81292	Enolase	extracellular; cytoplasm; cell surface
G0CYK9; embl-cds:AEG81301	Transcription elongation factor GreA	cytoplasm
G0CYM4; embl-cds:AEG81317	Fumarate hydratase class II	cytoplasm
G0CYQ3; embl-cds:AEG81345	Putative secreted protein	
G0CYQ7; embl-cds:AEG81349	Uncharacterized protein	
G0CYS8; embl-cds:AEG82468	Fructose-bisphosphate aldolase	cytoplasm
G0CYV5; embl-cds:AEG82498	Alcohol dehydrogenase	cytoplasm
G0CYV6; embl-cds:AEG82499	Aldehyde dehydrogenase	cytoplasm
G0CYW2; embl-cds:AEG82505	Chaperone protein DnaK	cytoplasm
G0CYY1; embl-cds:AEG82523	Urease accessory protein UreG	cytoplasm
G0CYY4; embl-cds:AEG82526	Urease subunit alpha	cytoplasm
G0CYY5; embl-cds:AEG82527	Urease subunit beta	cytoplasm
G0CZ22; embl-cds:AEG81380	2-oxoglutarate dehydrogenase E1 component	membrane
G0CZ53; embl-cds:AEG81411	Uncharacterized protein	
G0CZ62; embl-cds:AEG81420	Peptide chain release factor 1	cytoplasm
G0CZ71; embl-cds:AEG81429	ATP synthase subunit alpha	membrane
G0CZ73; embl-cds:AEG81431	ATP synthase subunit beta	membrane
G0CZ86; embl-cds:AEG81444	Electron transfer flavoprotein beta subunit	redox chain
G0CZ94; embl-cds:AEG82552	Phosphoenolpyruvate carboxykinase (GTP)	cytoplasm
G0CZA6; embl-cds:AEG82564	Putative secreted protein	
G0CZA9; embl-cds:AEG82567	Trehalose corynomycolyl transferase	cytoplasm
G0CZB9; embl-cds:AEG82576	UDP-galactopyranose mutase	cytoplasm
G0CZC6; embl-cds:AEG82583	Serine-tRNA ligase	cytoplasm
G0CZC8; embl-cds:AEG82585	Putative secreted protein	
G0CZG8; embl-cds:AEG82621	Uncharacterized protein	
G0CZP6; embl-cds:AEG81523	30S ribosomal protein S1	ribosome
G0CZQ1; embl-cds:AEG81528	Uncharacterized protein	cytoplasm
G0CZR5; embl-cds:AEG82638	30S ribosomal protein S6	ribosome

**Table 2:** Identification of annotated virulence factors in the exoproteome and surface-located proteome of *C. ulcerans*. Localization of the observed proteins from strains 809 and BR-AD22 as well as presence of an LPxTG motif for cell wall anchoring is indicated.

Name (NCBI)	Gene	Uniprot Identifier		Localization of Protein				LPxTG Motif
				Supernatant		Cell Surface		
		809	BR-AD22	809	BR-AD22	809	BR-AD22	
Putative ribosome binding protein	*rbp*	AEG80717	-	-	-	-	-	none
Corynebacterial protease CP40 precursor	*cpp*	AEG82501	AEG84830	+	+	-	-	none
Phospholipase D	*pld*	AEG80581	AEG82759	-	+	-	-	none
Surface-anchored protein, fimbrial subunit	*spaF*	AEG82476	AEG84808	+	+	+	+	LPKTG
Surface-anchored protein, fimbrial subunit	*spaE*	AEG82477	AEG84809	-	-	-	-	LPLTG
Surface-anchored protein, fimbrial subunit	*spaD*	AEG82479	AEG84811	+	+	-	-	LPMTG
Surface-anchored protein, fimbrial subunit	*spaC*	AEG82506	AEG84835	+	+	+	+	LPLTG
Surface-anchored protein, fimbrial subunit	*spaB*	AEG82507	AEG84836	+	+	-	-	LARTG
Resuscitation-promoting factor interacting protein	*rpfI*	AEG81666	AEG83858	+	+	+	+	none
Cell wall-associated hydrolase	*cwlH*	AEG82053	AEG84247	-	+	-	+	none
Sialidase precursor	*nanH*	AEG80974	AEG83155	+	+	+	+	none
Venom serine protease KN13	*vsp1*	AEG81049	AEG83233	+	+	+	+	none
Venom serine protease 2A	*vsp2*	AEG82491	-	-	-	-	-	none
Trypsin-like serine protease	*tspA*	AEG82376	AEG84712	+	+	-	-	none

## Supplementary Materials

The following are available online at http://www.mdpi.com/2227-7382/6/2/18/s1, Table S1: List of proteins identified as secretome proteins of *C. ulcerans* 809 and BR-AD22, Table S2: List of proteins identified as cell surface proteins of *C. ulcerans* 809 and BR-AD22.
