# Supplementary material for: Surface and Extracellular Proteome of the Emerging Pathogen Corynebacterium ulcerans
Source: Proteomes. 2018 Apr 17;6(2):18. doi: 10.3390/proteomes6020018 (PMC6027474; doi:10.3390/proteomes6020018)
Supplement: Supplementary file 1 [file proteomes-06-00018-s001.pdf]

Supplementary tables

Supplementary table 1. List of proteins identified as secretome proteins of *C. ulcerans* 809 and BR-AD22. Accession numbers, description and identified peptides per sample.

Secretome proteins		*Corynebacterium ulcerans* 809			*Corynebacterium ulcerans* BR-AD22		
Accession	Description	Peptides 1	Peptides 2	Peptides 3	Peptides 1	Peptides 2	Peptides 3
embl-cds:AEG80554	Putative Secreted Protein	5	6	4			
embl-cds:AEG80559	Peptidyl-Prolyl Cis-Trans Isomerase	2	5	3			
embl-cds:AEG80585	Iron-Siderophore Binding Protein	4	4	2			
embl-cds:AEG80587	Serine/Threonine Protein Kinase	2	1	3			
embl-cds:AEG80589	Penicillin-Binding Protein	7	6	6			
embl-cds:AEG80629	Putative Secreted Protein	15	16	15			
embl-cds:AEG80637	Putative Secreted Protein	5	4	4			
embl-cds:AEG80639	1,4-Alpha-Glucan-Branching Enzyme	55	52	50			
embl-cds:AEG80685	Hypothetical Protein	4	4	5			
embl-cds:AEG80688	Arabinosyl Transferase	4	4	4			
embl-cds:AEG80701	Putative Secreted Protein	2	5	3			
embl-cds:AEG80713	Hypothetical Protein	5	3	4			
embl-cds:AEG80719	Putative Secreted Protein	2	1	1			
embl-cds:AEG80756	Putative Secreted Lpxtg Protein	11	5	12			
embl-cds:AEG80757	Putative Secreted Protein	47	38	45			
embl-cds:AEG80763	Putative Secreted Lpxtg Protein	21	20	25			
embl-cds:AEG80768	Putative Secreted Protein	8	9	11			
embl-cds:AEG80769	Laminin Subunit Beta-2	28	31	29			
embl-cds:AEG80781	Putative Secreted Protein	12	15	14			
embl-cds:AEG80785	Putative Secreted Protein	15	11	17			
embl-cds:AEG80821	Putative Secreted Protein	19	20	23			
embl-cds:AEG80850	Putative Secreted Protein	5	5	5			
embl-cds:AEG80871	Zinc Abc Transport System Substrate-Binding Protein	2	3	1			
embl-cds:AEG80904	Putative Secreted Protein	11	13	19			
embl-cds:AEG80907	Dna-Directed Rna Polymerase Beta' Subunit	1	3	4			

embl-cds:AEG80909	Putative Secreted Lpxtg Protein	2	4	5			
embl-cds:AEG80955	Non-Specific Acid Phosphatase	10	11	9			
embl-cds:AEG80962	Putative Secreted Protein	3	3	3			
embl-cds:AEG80972	Adenylate Kinase	2	2	2			
embl-cds:AEG80974	Sialidase Precursor	8	8	5			
embl-cds:AEG80975	Putative Secreted Protein	5	5	5			
embl-cds:AEG81006	Molecular Chaperone	1	1	3			
embl-cds:AEG81023	Putative Secreted Protein	2	2	4			
embl-cds:AEG81024	Hypothetical Protein	3	3	2			
embl-cds:AEG81027	Manganese Abc Transporter, Substrate-Binding Protein	4	3	2			
embl-cds:AEG81038	Cell-Surface Hemin Receptor	14	13	12			
embl-cds:AEG81039	Iron Abc Transport System Substrate-Binding Protein	2	5	4			
embl-cds:AEG81044	Isocitrate Dehydrogenase	3	4	3			
embl-cds:AEG81049	Venom Serine Protease Kn13	3	3	4			
embl-cds:AEG81065	Acyl-Coa Carboxylase Alpha Subunit	2	2	4			
embl-cds:AEG81067	Hypothetical Protein	2	2	2			
embl-cds:AEG81091	Putative Secreted Protein	13	9	10			
embl-cds:AEG81107	Putative Secreted Protein	4	4	4			
embl-cds:AEG81125	Putative Secreted Protein	2	4	4			
embl-cds:AEG81141	Putative Membrane Protein	12	10	8			
embl-cds:AEG81154	Putative Secreted Protein	9	8	9			
embl-cds:AEG81165	Ferric Anguibactin-Binding Protein	8	4	8			
embl-cds:AEG81202	Abc Transporter Solute-Binding Protein	2	1	3			
embl-cds:AEG81227	Glutamate Abc Transport System Substrate-Binding Protein	7	8	7			
embl-cds:AEG81241	Trypsin-Like Serine Protease	3	3	5			
embl-cds:AEG81259	Resuscitation-Promoting Factor	12	9	14			
embl-cds:AEG81313	Putative Secreted Protein	1	1	2			
embl-cds:AEG81319	Putative Secreted Protein	1	1	2			
embl-cds:AEG81338	Putative Secreted Protein	21	21	19			

embl-cds:AEG81348	Putative Secreted Protein	9	9	9			
embl-cds:AEG81350	Hypothetical Protein	1	1	1			
embl-cds:AEG81379	Putative Secreted Protein	2	1	1			
embl-cds:AEG81408	Putative Secreted Protein	2	1	2			
embl-cds:AEG81413	Arginyl-Trna Synthetase	1	1	2			
embl-cds:AEG81460	Putative Secreted Protein	10	15	17			
embl-cds:AEG81468	Putative Secreted Protein	5	8	9			
embl-cds:AEG81476	Putative Membrane-Anchored Protein	2	1	3			
embl-cds:AEG81479	Putative Secreted Protein	1	2	1			
embl-cds:AEG81518	Putative Secreted Protein	3	3	2			
embl-cds:AEG81556	Putative Secreted Protein	5	5	2			
embl-cds:AEG81573	Iron Abc Transport System Substrate-Binding Protein	3	4	4			
embl-cds:AEG81587	Putative Secreted Protein	4	4	2			
embl-cds:AEG81592	6-Phosphogluconate Dehydrogenase	3	1	1			
embl-cds:AEG81605	Putative Secreted Protein	4	2	4			
embl-cds:AEG81635	Aspartate Ammonia-Lyase	1	1	2			
embl-cds:AEG81645	Putative Secreted Protein	5	3	5			
embl-cds:AEG81666	Resuscitation-Promoting Factor Interacting Protein	20	23	20			
embl-cds:AEG81667	Hypothetical Protein	4	4	5			
embl-cds:AEG81668	Aconitase	7	8	6			
embl-cds:AEG81674	Putative Secreted Lpxtg Protein	8	6	8			
embl-cds:AEG81692	Hypothetical Protein	2	2	3			
embl-cds:AEG81709	Hypothetical Protein	3	6	4			
embl-cds:AEG81733	Transketolase	1	2	4			
embl-cds:AEG81734	Transaldolase	4	5	4			
embl-cds:AEG81740	Triosephosphate Isomerase	3	3	4			
embl-cds:AEG81741	Phosphoglycerate Kinase	5	4	2			
embl-cds:AEG81742	Glyceraldehyde 3-Phosphate Dehydrogenase	5	6	4			
embl-cds:AEG81794	Thiol Peroxidase	2	2	1			
embl-cds:AEG81795	Peptidyl-Prolyl Cis-Trans Isomerase	2	3	4			

embl-cds:AEG81801	Protein-Export Membrane Protein	2	2	4			
embl-cds:AEG81819	Putative Secreted Protein	2	2	2			
embl-cds:AEG81858	Phosphoenolpyruvate-Protein Phosphotransferase	1	1	1			
embl-cds:AEG81927	Penicillin-Binding Protein	10	7	10			
embl-cds:AEG81975	Hypothetical Protein	1	1	1			
embl-cds:AEG82025	Cell Division Protein	2	1	1			
embl-cds:AEG82034	Penicillin-Binding Protein	2	1	1			
embl-cds:AEG82046	Serine/Threonine Protein Kinase	8	10	10			
embl-cds:AEG82070	Dihydrolipoamide Acyltransferase	2	1	1			
embl-cds:AEG82080	Iron-Siderophore Binding Protein	2	1	1			
embl-cds:AEG82114	Pyruvate Dehydrogenase E1 Component	3	2	3			
embl-cds:AEG82129	Putative Secreted Lpxtg Protein	1	2	1			
embl-cds:AEG82165	Putative Secreted Protein	1	2	4			
embl-cds:AEG82175	Hypothetical Protein	3	2	2			
embl-cds:AEG82202	Nucleoside Diphosphate Kinase	1	1	1			
embl-cds:AEG82213	Trigger Factor	1	1	1			
embl-cds:AEG82238	Putative Secreted Protein	2	1	1			
embl-cds:AEG82239	Putative Secreted Protein	18	19	18			
embl-cds:AEG82244	Hypothetical Protein	2	3	4			
embl-cds:AEG82283	Protein Piccolo	14	11	14			
embl-cds:AEG82284	Hypothetical Protein	1	1	1			
embl-cds:AEG82285	Putative Secreted Protein	1	1	1			
embl-cds:AEG82299	Cysteine Synthase	4	3	4			
embl-cds:AEG82314	Phosphate Upatke System Phosphate-Binding Protein	3	1	1			
embl-cds:AEG82337	Phosphoribosylamine-Glycine Ligase	2	1	2			
embl-cds:AEG82344	Putative Secreted Protein	10	11	9			
embl-cds:AEG82349	Putative Secreted Lpxtg Protein	24	28	22			
embl-cds:AEG82363	Putative Secreted Protein	10	6	12			
embl-cds:AEG82375	Laminin Subunit Alpha-1	1	2	3			
embl-cds:AEG82376	Trypsin-Like Serine Protease	8	6	6			

embl-cds:AEG82395	D-Alanyl-D-Alanine Carboxypeptidase	6	5	5			
embl-cds:AEG82399	Putative Secreted Protein	12	11	11			
embl-cds:AEG82410	Hypothetical Protein	2	2	3			
embl-cds:AEG82411	Molecular Chaperone	5	2	2			
embl-cds:AEG82415	Putative Secreted Protein	21	26	22			
embl-cds:AEG82436	Acetate Kinase	3	2	4			
embl-cds:AEG82446	Putative Secreted Protein	3	4	4			
embl-cds:AEG82449	Hypothetical Protein	1	1	1			
embl-cds:AEG82454	Putative Secreted Protein	11	10	10			
embl-cds:AEG82460	Putative Secreted Protein	28	26	29			
embl-cds:AEG82467	Putative Secreted Protein	49	53	56			
embl-cds:AEG82476	Surface-Anchored Protein, Fimbrial Subunit	9	7	8			
embl-cds:AEG82479	Surface-Anchored Protein, Fimbrial Subunit	7	9	7			
embl-cds:AEG82485	Putative Secreted Protein	2	3	4			
embl-cds:AEG82493	Putative Membrane Protein	1	1	1			
embl-cds:AEG82495	Hypothetical Protein	15	20	18			
embl-cds:AEG82500	Putative Secreted Protein	4	3	3			
embl-cds:AEG82501	Corynebacterial Protease Cp40 Precursor	4	9	6			
embl-cds:AEG82506	Surface-Anchored Protein, Fimbrial Subunit	39	41	43			
embl-cds:AEG82513	Hypothetical Protein	7	4	5			
embl-cds:AEG82521	Abc Transport System, Substrate-Binding Protein	7	8	7			
embl-cds:AEG82544	Hypothetical Protein	7	7	5			
embl-cds:AEG82558	Putative Secreted Protein	7	5	5			
embl-cds:AEG82562	Acyl-Coa Synthetase	11	9	9			
embl-cds:AEG82563	Envelope Lipids Regulation Factor	2	1	2			
embl-cds:AEG82565	Trehalose Corynomycolyl Transferase	45	45	46			
embl-cds:AEG82574	Glycerophosphoryl Diester Phosphodiesterase	12	8	12			
embl-cds:AEG82580	Putative Secreted Protein	17	19	18			
embl-cds:AEG82593	Cell-Surface Hemin Receptor	1	1	2			
embl-cds:AEG82616	Housekeeping Sortase	4	3	3			

embl-cds:AEG82641	Penicillin-Binding Protein	8	8	6			
embl-cds:AEG82649	Cell-Surface Hemin Receptor	5	2	6			
embl-cds:AEG82658	Putative Secreted Protein	5	3	2			
embl-cds:AEG82663	Putative Secreted Lpxtg Protein	1	1	1			
embl-cds:AEG82674	Putative Secreted Protein	4	4	3			
embl-cds:AEG82677	4-Aminobutyrate Aminotransferase	1	1	2			
embl-cds:AEG82701	Putative Secreted Protein	1	1	1			
embl-cds:AEG82709	Putative Secreted Protein	1	4	5			
embl-cds:AEG82712	Thioredoxin Reductase	1	2	1			
G0CTC1; embl-cds:AEG81546	Argininosuccinate Synthase				3	4	6
G0CTC4; embl-cds:AEG81549	Tyrosine--Trna Ligase				3	1	2
G0CTF8; embl-cds:AEG81583	Inhibitor Of Odh Activity				1	4	4
G0CTH0; embl-cds:AEG81595	Uncharacterized Protein				1	1	2
G0CTI2; embl-cds:AEG81607	Rna Polymerase-Binding Protein Rbpa				1	1	2
G0CTJ1; embl-cds:AEG82717	Chromosome Partitioning Protein				1	1	2
G0CTN4; embl-cds:AEG81649	Uncharacterized Protein				1	1	3
G0CTQ7; embl-cds:AEG81672	Upf0210 Protein Culc22_01154				1	1	6
G0CTS2; embl-cds:AEG81687	Uncharacterized Protein	1	2	2	1		
G0CTS8; embl-cds:AEG81693	Uncharacterized Protein	4	3	3			1
G0CTS9; embl-cds:AEG81694	Uncharacterized Protein	2	2	2			1
G0CTU2; embl-cds:AEG81708	Uncharacterized Protein	3	2	3	1		
G0CTV7; embl-cds:AEG81724	Abc-Type Transport System Involved In Fe-S Cluster Assembly Atp-Binding Protein			1	3	5	6
G0CTV8; embl-cds:AEG81725	Abc-Type Transport System Involved In Fe-S Cluster Assembly Permease Component	1	1	1	7	6	7

G0CTV9; embl-cds:AEG81726	Abc-Type Transport System Involved In Fe-S Cluster Assembly Permease Component				7	7	11
G0CTY5; embl-cds:AEG81752	Ribulose-Phosphate 3-Epimerase				1	1	1
G0CTZ3; embl-cds:AEG81759	Dna-Directed Rna Polymerase Subunit Omega				2	1	2
G0CU06; embl-cds:AEG81772	Elongation Factor Ef-P		2	2	2	4	4
G0CU14; embl-cds:AEG81780	Putative Secreted Protein	5	5	6	5	7	10
G0CU23; embl-cds:AEG80551	Dna Gyrase Subunit A				7	5	3
G0CU44; embl-cds:AEG81799	Putative Secreted Protein	6	1	6	3	6	7
G0CU51; embl-cds:AEG81806	Probable Transcriptional Regulatory Protein Culc22_01288				1	3	2
G0CU61; embl-cds:AEG81816	Threonine--Trna Ligase			2	3	5	5
G0CU76; embl-cds:AEG81831	Deoxyuridine 5'-Triphosphate Nucleotidohydrolase				2	2	1
G0CU78; embl-cds:AEG81833	Uncharacterized Protein				1	1	1
G0CU89; embl-cds:AEG81844	Dtxr-Family Transcription Regulator	1	1	1	2	6	7
G0CU95; embl-cds:AEG81850	Alkyl Hydroperoxide Reductase	2		3	3	8	7
G0CUC6; embl-cds:AEG80578	Uncharacterized Protein	1			1	2	4
G0CUG4; embl-cds:AEG80613	Uncharacterized Protein		1	1	1	1	1
G0CUI8; embl-cds:AEG81863	Phosphocarrier Protein Hpr	2	2	3			
G0CUJ6; embl-cds:AEG81871	Diaminopimelate Epimerase				1	3	3
G0CUM1; embl-cds:AEG81896	Polyribonucleotide Nucleotidyltransferase	4	2	5	13	10	12
G0CUN1; embl-cds:AEG81906	Translation Initiation Factor If-2				4	9	7
G0CUN3; embl-cds:AEG81908	Transcription Termination/Antitermination Protein Nusa				5	5	4

G0CUP9; embl-cds:AEG81923	Mycothiol Reductase				6	6	4
G0CUQ4; embl-cds:AEG81928	4-Hydroxy-3-Methylbut-2-En-1-Yl Diphosphate Synthase (Flavodoxin)				3	3	6
G0CUR3; embl-cds:AEG81937	Elongation Factor Ts	6	3	6	9	12	17
G0CUR4; embl-cds:AEG81938	30S Ribosomal Protein S2				3	1	3
G0CUV7; embl-cds:AEG80677	Upf0145 Protein Culc22_00134				3	3	2
G0CUW8; embl-cds:AEG80687	Putative Secreted Protein	3	3	5	6	5	3
G0CUX1; embl-cds:AEG80690	Decaprenylphosphoryl-Beta-D-Ribose 2-Epimerase Component				3	3	4
G0CUX8; embl-cds:AEG80697	Uncharacterized Protein	1	1	1		1	1
G0CV12; embl-cds:AEG81955	50S Ribosomal Protein L19				2	2	3
G0CV22; embl-cds:AEG81965	Nitrogen Regulatory Protein P-Ii				1	1	1
G0CV41; embl-cds:AEG81983	Putative Secreted Protein	4	4	4	4	5	5
G0CV55; embl-cds:AEG81997	Histidinol Dehydrogenase				1	2	1
G0CV58; embl-cds:AEG82000	Putative Secreted Protein	2	2	4	3	2	4
G0CV74; embl-cds:AEG82016	Putative Secreted Protein	2	2	2	2	1	2
G0CV86; embl-cds:AEG80721	Pyridoxal 5'-Phosphate Synthase Subunit Pdxs			1	4	5	5
G0CV98; embl-cds:AEG80733	Uncharacterized Protein				4	6	5
G0CVA1; embl-cds:AEG80736	Nucleoid-Associated Protein Culc22_00193	1		1	2	2	2
G0CVA8; embl-cds:AEG80743	Putative Secreted Protein	11	10	10	6	6	7
G0CVB1; embl-cds:AEG80746	Uncharacterized Protein				1	1	2
G0CVC7; embl-cds:AEG80762	Aspartokinase		1		2	5	7

G0CVC9; embl-cds:AEG80764	Aspartate-Semialdehyde Dehydrogenase				3	2	4
G0CVD1; embl-cds:AEG80766	Catalase	1	1	4	10	14	14
G0CVF7; embl-cds:AEG80788	Uncharacterized Protein				3	2	1
G0CVF9; embl-cds:AEG80790	Crp-Family Transcription Regulator				2	5	6
G0CVG4; embl-cds:AEG80795	Putative Membrane Protein	2	1	1	1	2	2
G0CVI6; embl-cds:AEG82045	Uncharacterized Protein				1	2	2
G0CVJ8; embl-cds:AEG82057	Ubiquinol-Cytochrome C Reductase Cytochrome C Subunit	2	1	1	2	1	2
G0CVK1; embl-cds:AEG82060	Cytochrome C Oxidase Subunit Ii	6	5	5	6	5	7
G0CVK3; embl-cds:AEG82062	Uncharacterized Protein				2	1	2
G0CVK8; embl-cds:AEG82067	Branched-Chain-Amino-Acid Aminotransferase		1	1	4	4	6
G0CVM9; embl-cds:AEG82088	Uncharacterized Protein				1	2	2
G0CVR2; embl-cds:AEG80808	Cold Shock-Like Protein A	1	1	2	3	4	4
G0CVS1; embl-cds:AEG80815	Putative Secreted Protein	12	12	11	13	11	11
G0CVT0; embl-cds:AEG80823	Dihydrolipoyl Dehydrogenase	1	1		8	7	10
G0CVT3; embl-cds:AEG80826	Succinate Dehydrogenase Flavoprotein Subunit				2	2	2
G0CVT4; embl-cds:AEG80827	Succinate Dehydrogenase Iron-Sulfur Protein				1	2	1
G0CVT9; embl-cds:AEG80832	Putative Secreted Protein	4	3	2	5	6	4
G0CVU1; embl-cds:AEG80834	Formate Acetyltransferase				1	2	1
G0CVU2; embl-cds:AEG80835	Formate Acetyltransferase				3	4	4
G0CVU4; embl-cds:AEG80837	Uncharacterized Protein	2	2	2	2	3	3

G0CVU8; embl-cds:AEG80841	2,3-Bisphosphoglycerate-Dependent Phosphoglycerate Mutase		1		6	7	6
G0CVY9; embl-cds:AEG82113	Uncharacterized Protein				2	3	2
G0CW00; embl-cds:AEG82124	Glutamine--Fructose-6-Phosphate Aminotransferase [Isomerizing]	1		1	3	9	10
G0CW25; embl-cds:AEG82149	4-Alpha-Glucanotransferase				3	6	7
G0CW81; embl-cds:AEG80892	50S Ribosomal Protein L11			1	1	1	1
G0CW86; embl-cds:AEG80897	50S Ribosomal Protein L10		1	1	3	2	4
G0CW87; embl-cds:AEG80898	50S Ribosomal Protein L7/L12	2	1	1	5	3	4
G0CW95; embl-cds:AEG80906	Dna-Directed Rna Polymerase Subunit Beta	2		1	17	10	11
G0CWB0; embl-cds:AEG80921	Uncharacterized Protein				1	2	1
G0CWB2; embl-cds:AEG80923	30S Ribosomal Protein S7				4	4	3
G0CWB3; embl-cds:AEG80924	Elongation Factor G	11	13	10	26	25	27
G0CWB4; embl-cds:AEG80925	Elongation Factor Tu	14	11	10	20	22	23
G0CWC4; embl-cds:AEG80935	50S Ribosomal Protein L3	2	3	3	4	5	5
G0CWC5; embl-cds:AEG80936	50S Ribosomal Protein L4	4	2	4	4	7	9
G0CWC6; embl-cds:AEG80937	50S Ribosomal Protein L23	1	2	1	4	3	4
G0CWC7; embl-cds:AEG80938	50S Ribosomal Protein L2	2	5	4	7	8	10
G0CWC8; embl-cds:AEG80939	30S Ribosomal Protein S19				4	5	4
G0CWC9; embl-cds:AEG80940	50S Ribosomal Protein L22	1			3	4	3
G0CWD0; embl-cds:AEG80941	30S Ribosomal Protein S3	1			4	7	5
G0CWD1; embl-cds:AEG80942	50S Ribosomal Protein L16			1	2	2	3

G0CWD3; embl-cds:AEG80944	30S Ribosomal Protein S17				2	1	3
G0CWD4; embl-cds:AEG80945	Putative Secreted Protein			1	13	19	18
G0CWD9; embl-cds:AEG80950	50S Ribosomal Protein L24	1	1	1	2	2	4
G0CWE0; embl-cds:AEG80951	50S Ribosomal Protein L5				3	3	4
G0CWE5; embl-cds:AEG80956	30S Ribosomal Protein S8				1	1	1
G0CWJ7; embl-cds:AEG82180	Elongation Factor 4				2	4	4
G0CWL4; embl-cds:AEG82197	Gtpase Obg				1	3	1
G0CWL5; embl-cds:AEG82198	50S Ribosomal Protein L27	1	1		3	3	1
G0CWL6; embl-cds:AEG82199	50S Ribosomal Protein L21	1			2	3	2
G0CWM4; embl-cds:AEG82207	Malate Dehydrogenase				2	2	5
G0CWM5; embl-cds:AEG82208	Tetr-Family Transcription Regulator				3	3	2
G0CWM9; embl-cds:AEG82211	Atp-Dependent Clp Protease Proteolytic Subunit	1			3	2	3
G0CWN2; embl-cds:AEG80960	50S Ribosomal Protein L30			1	1	2	2
G0CWN3; embl-cds:AEG80961	50S Ribosomal Protein L15		1	2	3	5	4
G0CWN5; embl-cds:AEG80963	Maltotriose-Binding Protein	9	11	9	11	9	10
G0CWP4; embl-cds:AEG80973	Methionine Aminopeptidase				2	2	1
G0CWP7; embl-cds:AEG80976	Translation Initiation Factor If-1	1	1	1	1	2	
G0CWQ0; embl-cds:AEG80979	30S Ribosomal Protein S4				5	2	4
G0CWQ1; embl-cds:AEG80980	Dna-Directed Rna Polymerase Subunit Alpha	3	2	3	8	10	12
G0CWR4; embl-cds:AEG80992	30S Ribosomal Protein S9				4	3	4

G0CWS7; embl-cds:AEG81005	10 Kda Chaperonin		1	2	2	4	4
G0CWT5; embl-cds:AEG81012	Uncharacterized Protein				2	6	6
G0CWU3; embl-cds:AEG81019	Putative Secreted Protein	3	4	2	5	2	2
G0CWX0; embl-cds:AEG82217	Ribose 5-Phosphate Isomerase				2	3	2
G0CWX7; embl-cds:AEG82224	Uncharacterized Protein				1	1	1
G0CX22; embl-cds:AEG82249	Bacterioferritin Comigratory Protein				1	2	4
G0CX62; embl-cds:AEG81057	Uracil Phosphoribosyltransferase				1	1	2
G0CX66; embl-cds:AEG81061	Dihydrolipoamide Dehydrogenase		1	1	3	5	5
G0CX81; embl-cds:AEG81075	Acyl-Coa Carboxylase Complex Subunit				1	5	3
G0CX97; embl-cds:AEG81093	Lytr-Family Transcription Regulator	7	2	5	4	6	8
G0CX99; embl-cds:AEG81095	Mannose-1-Phosphate Guanyltransferase				1	1	5
G0CXB0; embl-cds:AEG81105	Two-Component System Transcriptional Regulatory Protein				1	2	1
G0CXB7; embl-cds:AEG81112	Uncharacterized Protein				1	3	5
G0CXC9; embl-cds:AEG81124	Putative Secreted Protein	13	11	11	11	10	11
G0CXD3; embl-cds:AEG82274	Ribonucleoside-Diphosphate Reductase Subunit Beta	1	1		5	7	5
G0CXD5; embl-cds:AEG82276	Ribonucleoside-Diphosphate Reductase			2	8	10	11
G0CXE0; embl-cds:AEG82281	Uncharacterized Protein				1	4	1
G0CXF5; embl-cds:AEG82295	Udp-N-Acetylglucosamine 1-Carboxyvinyltransferase				3	2	4
G0CXF7; embl-cds:AEG82297	Luxr-Family Transcription Regulator				1	1	1
G0CXG7; embl-cds:AEG82307	Succinyl-Coa:Coenzyme A Transferase	4	3	7	17	18	20

G0CXH8; embl-cds:AEG82318	Upf0678 Fatty Acid-Binding Protein-Like Protein Culc22_01893				6	3	5
G0CXI9; embl-cds:AEG82329	Phosphoribosylformylglycinamidine Synthase Subunit Purq				2	3	2
G0CXJ3; embl-cds:AEG82333	Phosphoribosylaminoimidazole-Succinocarboxamide Synthase				2	1	2
G0CXK1; embl-cds:AEG82341	Two-Component System Transcriptional Regulatory Protein				2	4	3
G0CXK8; embl-cds:AEG81127	Uncharacterized Protein	1	1	1	3	3	3
G0CXK9; embl-cds:AEG81128	Uncharacterized Protein	2	1	2	2	3	2
G0CXM1; embl-cds:AEG81140	Uncharacterized Protein	1	1		1	1	1
G0CXN2; embl-cds:AEG81150	Oxaloacetate Decarboxylase Alpha Subunit				1	2	3
G0CXP5; embl-cds:AEG81163	Carbonic Anhydrase	4	4	4	6	6	7
G0CXQ6; embl-cds:AEG81174	Resuscitation-Promoting Factor	12	10	13	12	12	12
G0CXQ9; embl-cds:AEG81178	Putative Secreted Protein	1	3	1	2	1	3
G0CXR6; embl-cds:AEG81185	Citrate Synthase		1		6	8	7
G0CXR9; embl-cds:AEG81188	Superoxide Dismutase [Cu-Zn]	2	2	2	2	3	4
G0CY06; embl-cds:AEG82362	Putative Secreted Protein	4	4	3	6	3	3
G0CY09; embl-cds:AEG82365	Putative Secreted Protein	1	1	3	3	6	3
G0CY30; embl-cds:AEG81210	Uncharacterized Protein				1	1	2
G0CY42; embl-cds:AEG81222	Uncharacterized Protein	5	4	4	6	5	6
G0CY56; embl-cds:AEG81236	50S Ribosomal Protein L28		1		1	2	2
G0CY58; embl-cds:AEG81238	50S Ribosomal Protein L32	1			1	2	1
G0CY59; embl-cds:AEG81239	Two-Component System Transcriptional Regulatory Protein	3	2	3	5	6	6

G0CY67; embl-cds:AEG81247	Utp--Glucose-1-Phosphate Uridylyltransferase				1	1	1
G0CY83; embl-cds:AEG81263	Uncharacterized Protein				5	6	4
G0CYA4; embl-cds:AEG81281	50S Ribosomal Protein L25		1	1	2	3	3
G0CYC4; embl-cds:AEG82393	Hypoxanthine Phosphoribosyltransferase	1	1		2	3	3
G0CYC7; embl-cds:AEG82396	Inorganic Pyrophosphatase				5	5	4
G0CYH1; embl-cds:AEG82442	Purine Phosphoribosyltransferase	1	1	2	2	5	4
G0CYH2; embl-cds:AEG82443	Putative Membrane Protein	10	10	9	8	8	13
G0CYI6; embl-cds:AEG82457	Adenylosuccinate Synthetase				1	1	2
G0CYJ1; embl-cds:AEG81283	Ribose-Phosphate Pyrophosphokinase			1	4	6	7
G0CYK0; embl-cds:AEG81292	Enolase	3	2	2	11	8	12
G0CYK9; embl-cds:AEG81301	Transcription Elongation Factor Grea	1		1	3	3	4
G0CYM4; embl-cds:AEG81317	Fumarate Hydratase Class Ii	2	2	2	13	12	11
G0CYQ3; embl-cds:AEG81345	Putative Secreted Protein	5	4	6	5	5	5
G0CYQ5; embl-cds:AEG81347	Uncharacterized Protein				2	2	6
G0CYQ7; embl-cds:AEG81349	Uncharacterized Protein	1	1	2	9	4	14
G0CYS8; embl-cds:AEG82468	Fructose-Bisphosphate Aldolase	2	2		9	10	10
G0CYV5; embl-cds:AEG82498	Alcohol Dehydrogenase		2	1	6	6	6
G0CYV6; embl-cds:AEG82499	Aldehyde Dehydrogenase	5	3	5	3	9	6
G0CYW2; embl-cds:AEG82505	Chaperone Protein Dnak	6	5	4	20	17	22
G0CYY1; embl-cds:AEG82523	Urease Accessory Protein Ureg	1			5	5	4

G0CYY4; embl-cds:AEG82526	Urease Subunit Alpha	5	5	7	9	9	8
G0CYY5; embl-cds:AEG82527	Urease Subunit Beta	3	2	4	4	5	5
G0CYY6; embl-cds:AEG82528	Urease Subunit Gamma				2	2	3
G0CYZ4; embl-cds:AEG82536	Deoxycytidine Triphosphate Deaminase				1	1	1
G0CZ22; embl-cds:AEG81380	2-Oxoglutarate Dehydrogenase E1 Component	1	1		8	12	12
G0CZ35; embl-cds:AEG81393	Atp-Dependent Rna Helicase Dead				2	3	4
G0CZ53; embl-cds:AEG81411	Uncharacterized Protein			1	6	7	6
G0CZ57; embl-cds:AEG81415	Homoserine Dehydrogenase				5	5	5
G0CZ62; embl-cds:AEG81420	Peptide Chain Release Factor 1	1			4	4	4
G0CZ71; embl-cds:AEG81429	Atp Synthase Subunit Alpha	1		2	6	8	9
G0CZ73; embl-cds:AEG81431	Atp Synthase Subunit Beta		2	2	14	11	9
G0CZ78; embl-cds:AEG81436	Uncharacterized Protein	1			3	3	3
G0CZ83; embl-cds:AEG81441	Uncharacterized Protein				2	3	1
G0CZ86; embl-cds:AEG81444	Electron Transfer Flavoprotein Beta Subunit			1	4	2	2
G0CZ94; embl-cds:AEG82552	Phosphoenolpyruvate Carboxykinase [Gtp]	1	3	2	13	16	14
G0CZ95; embl-cds:AEG82553	Trna (Guanine-N(7)-)-Methyltransferase	1	1	2		3	2
G0CZA6; embl-cds:AEG82564	Putative Secreted Protein	1	1	1	2	2	2
G0CZA9; embl-cds:AEG82567	Trehalose Corynomycolyl Transferase	6	6	10	10	8	10
G0CZB9; embl-cds:AEG82576	Udp-Galactopyranose Mutase	1		1	5	7	7
G0CZC6; embl-cds:AEG82583	Serine--Trna Ligase	1		1	5	7	5

G0CZC8; embl-cds:AEG82585	Putative Secreted Protein	2	4	3	5	3	3
G0CZG8; embl-cds:AEG82621	Uncharacterized Protein		1		2	4	5
G0CZI7; embl-cds:AEG81463	Aspartyl/Glutamyl-Trna(Asn/Gln) Amidotransferase Subunit B				5	5	9
G0CZI8; embl-cds:AEG81464	Uncharacterized Protein				1	4	5
G0CZP6; embl-cds:AEG81523	30S Ribosomal Protein S1	7	7	6	18	15	15
G0CZQ1; embl-cds:AEG81528	Uncharacterized Protein			1	2	3	1
G0CZQ5; embl-cds:AEG82627	Rod-Shaped Morphology Protein				3	2	2
G0CZQ6; embl-cds:AEG82628	Thioredoxin	1			2	3	4
G0CZR3; embl-cds:AEG82636	50S Ribosomal Protein L9				2	2	2
G0CZR5; embl-cds:AEG82638	30S Ribosomal Protein S6	1	1	1	3	4	4
G0CZU4; embl-cds:AEG82666	N-Acylglucosamine-6-Phosphate 2-Epimerase				1	4	3
G0CZU7; embl-cds:AEG82669	Putative Secreted Protein	7	5	4	6	6	10
G0CTB4	Phenylalanine--Trna Ligase Beta Subunit				6	9	10
G0CTB5	N-Acetyl-Gamma-Glutamyl-Phosphate Reductase				1	1	1
G0CTC2	Argininosuccinate Lyase				1	2	2
G0CTC6	Uncharacterized Protein				2	2	2
G0CTD1	Putative Secreted Protein				1	2	3
G0CTG2	Putative Secreted Protein				2	3	1
G0CTG7	6-Phosphogluconate Dehydrogenase, Decarboxylating				6	5	8
G0CTH4	Nadh Dehydrogenase				6	4	4
G0CTI0	Putative Secreted Protein				2	3	2
G0CTI6	Cobaltochelatase				7	11	13
G0CTL9	Formate--Tetrahydrofolate Ligase				4	3	6
G0CTM0	Aspartate Ammonia-Lyase				6	5	6
G0CTM7	L-Cysteine:1D-Myo-Inositol 2-Amino-2-Deoxy-Alpha-D-Glucopyranoside Ligase				3	3	3
G0CTN0	Putative Secreted Protein				3	4	5
G0CTQ1	Resuscitation-Promoting Factor Interacting Protein				18	18	15

G0CTQ3	Aconitase				15	17	17
G0CTR0	Putative Secreted Lpxtg Protein				9	11	7
G0CTV6	Cysteine Desulfurase				5	6	9
G0CTW6	Transketolase				13	13	14
G0CTW7	Transaldolase				6	6	6
G0CTX0	6-Phosphogluconolactonase				2	4	6
G0CTX3	Triosephosphate Isomerase				7	5	5
G0CTX4	Phosphoglycerate Kinase				11	15	13
G0CTX5	Glyceraldehyde-3-Phosphate Dehydrogenase				8	11	10
G0CTY2	Riboflavin Biosynthesis Protein Ribba				1	4	3
G0CTZ1	S-Adenosylmethionine Synthase				2	4	5
G0CTZ4	Guanylate Kinase				1	2	2
G0CTZ7	Carbamoyl-Phosphate Synthase Large Chain				1	2	1
G0CTZ8	Carbamoyl-Phosphate Synthase Small Chain				2	2	5
G0CU07	Xaa-Pro Aminopeptidase				4	2	2
G0CU16	Dna Polymerase Iii Subunit Beta				2	5	4
G0CU26	Alanine--Trna Ligase				1	4	5
G0CU29	Aspartyl-Trna Synthetase				9	10	9
G0CU36	L-Serine Dehydratase				4	5	5
G0CU39	Probable Thiol Peroxidase				5	6	5
G0CU40	Peptidyl-Prolyl Cis-Trans Isomerase				2	3	4
G0CU46	Protein Translocase Subunit Secd				3	3	4
G0CU62	Putative Secreted Protein				1	4	3
G0CU63	Putative Secreted Protein				3	4	7
G0CU64	Putative Secreted Protein				2	1	2
G0CU69	Uncharacterized Protein				1	6	6
G0CU75	Uncharacterized Protein				3	3	5
G0CU90	Udp-Glucose 4-Epimerase				6	8	7
G0CUA4	Putative Secreted Protein				7	8	6
G0CUA9	Peptidyl-Prolyl Cis-Trans Isomerase				6	6	7
G0CUC9	Phospholipase D				7	7	10
G0CUD3	Iron-Siderophore Binding Protein				8	5	6
G0CUD5	Serine/Threonine Protein Kinase				2	4	3
G0CUD6	Serine/Threonine Protein Kinase				3	2	1
G0CUE4	Putative Secreted Protein				2	1	2
G0CUF8	Uncharacterized Protein				2	1	2
G0CUI2	Putative Secreted Protein				3	6	3
G0CUI4	Phosphoenolpyruvate-Protein Phosphotransferase				6	10	9
G0CUK2	Trna-2-Methylthio-N(6)-Dimethylallyladenosine Synthase				1	1	3
G0CUM3	Inosine-Uridine Preferring Nucleoside Hydrolase				1	2	2
G0CUN5	Putative Secreted Protein				1	1	1

G0CUN6	Proline--Trna Ligase				9	6	11
G0CUQ2	Methionine Aminopeptidase				1	1	2
G0CUQ3	Penicillin-Binding Protein				6	10	6
G0CUR9	1,4-Alpha-Glucan-Branching Enzyme				40	44	53
G0CUS0	Putative Secreted Protein				13	14	13
G0CUU7	Putative Secreted Protein				4	4	3
G0CUW2	Glycerol Kinase				3	5	5
G0CUW5	Probable Zinc Metalloprotease				4	4	11
G0CUW6	Uncharacterized Protein				4	3	2
G0CUW9	Arabinosyl Transferase				6	5	5
G0CUX2	Decaprenylphosphoryl-Beta-D-Ribose 2-Epimerase Component				4	2	5
G0CUY8	Putative Phenylalanine Aminotransferase				2	2	3
G0CUZ3	Uncharacterized Protein				7	5	8
G0CUZ9	Putative Secreted Protein				1	3	1
G0CV17	Ribosome Maturation Factor Rimm				1	1	1
G0CV19	30S Ribosomal Protein S16				2	2	3
G0CV32	Uncharacterized Protein				1	1	3
G0CV33	Alanine Dehydrogenase				1	1	2
G0CV40	Alpha-1,4 Glucan Phosphorylase				1	7	8
G0CV42	Pyruvate Kinase				6	7	9
G0CV73	Putative Secreted Protein				4	10	6
G0CV77	Isoleucine--Trna Ligase				9	15	13
G0CV81	Cell Division Protein Sepf				2	3	5
G0CVB9	Deoxyribose-Phosphate Aldolase				1	1	1
G0CVC1	Putative Secreted Lpxtg Protein				4	4	3
G0CVC2	Putative Secreted Protein				11	8	8
G0CVC8	Putative Secreted Lpxtg Protein				27	20	19
G0CVD2	Uncharacterized Protein				18	22	24
G0CVD3	Putative Secreted Protein				12	11	11
G0CVD4	Laminin Subunit Beta-2				30	23	27
G0CVF2	Putative Secreted Protein				14	16	17
G0CVF4	Putative Secreted Protein				12	17	21
G0CVG7	Cell Division Protein				1	1	1
G0CVG8	Udp-N-Acetylmuramate--L-Alanine Ligase				4	6	5
G0CVH4	Udp-N-Acetylmuramyl-Tripeptide Synthetase				2	3	3
G0CVH5	Penicillin-Binding Protein				2	4	4
G0CVI7	Serine/Threonine Protein Kinase				8	10	9
G0CVI8	Phospho-2-Dehydro-3-Deoxyheptonate Aldolase				2	3	5
G0CVJ4	Cell Wall-Associated Hydrolase				5	5	10
G0CVJ5	Putative Secreted Protein				1	2	2
G0CVK2	Asparagine Synthetase				1	1	1
G0CVK5	Putative Cobinamide Kinase/Cobinamide Phosphate Guanylyltransferase				1	1	1

G0CVK9	Probable Cytosol Aminopeptidase				3	4	1
G0CVL1	Dihydrolipoamide Acyltransferase				7	5	9
G0CVL2	Glycine Dehydrogenase (Decarboxylating)				4	6	6
G0CVL3	Aminomethyltransferase				4	6	5
G0CVL4	Glycine Cleavage System H Protein				2	1	1
G0CVM5	Glutamine Synthetase				1	5	4
G0CVN4	Threonine Synthase				1	4	5
G0CVP4	Galactokinase				1	3	1
G0CVS6	Uncharacterized Protein				1	5	3
G0CVS7	Putative Secreted Protein				20	22	24
G0CVU5	Udp-N-Acetylenolpyruvoylglucosamine Reductase				2	3	3
G0CVU6	Uncharacterized Protein				5	3	6
G0CVV7	Putative Secreted Protein				2	3	3
G0CVW3	Porphobilinogen Deaminase				2	4	3
G0CVZ0	Pyruvate Dehydrogenase E1 Component				11	11	16
G0CVZ8	Thiamine Biosynthesis Protein				1	2	3
G0CW01	Putative Secreted Protein				1	1	3
G0CW05	Putative Secreted Lpxtg Protein				2	2	2
G0CW08	Glycine--Trna Ligase				9	14	11
G0CW15	Pyridoxamine Kinase				3	2	3
G0CW28	Peptidyl-Dipeptidase				6	7	11
G0CW41	Putative Secreted Protein				6	5	6
G0CW50	Uncharacterized Protein				3	9	7
G0CW57	Uncharacterized Protein				4	1	3
G0CW66	Uncharacterized Protein				4	5	4
G0CW80	Transcription Termination/Antitermination Protein Nusg				5	6	4
G0CW88	Putative Secreted Lpxtg Protein				1	3	2
G0CW93	Putative Secreted Protein				19	12	13
G0CW96	Dna-Directed Rna Polymerase				24	25	31
G0CW98	Putative Secreted Lpxtg Protein				3	6	7
G0CWA6	Putative Membrane Protein				3	4	4
G0CWE4	Non-Specific Acid Phosphatase				13	15	14
G0CWE6	50S Ribosomal Protein L6				4	4	4
G0CWF2	Uncharacterized Protein				3	5	4
G0CWF3	Uncharacterized Protein				8	3	5
G0CWF8	Uncharacterized Protein				10	12	12
G0CWF9	Uncharacterized Protein				1	1	3
G0CWH0	Putative Secreted Protein				7	6	6
G0CWI8	Single-Stranded Dna-Binding Protein				1	2	3
G0CWK7	Ribosomal Silencing Factor Rsfs				2	1	1
G0CWL0	Uncharacterized Protein				1	3	4
G0CWL9	Nucleoside Diphosphate Kinase				3	3	5
G0CWM3	Valine--Trna Ligase				9	8	13

G0CWN1	Trigger Factor				6	7	10
G0CWN4	Putative Secreted Protein				2	2	1
G0CWP3	Adenylate Kinase				5	6	8
G0CWP5	Sialidase				17	14	16
G0CWP6	Putative Secreted Protein				7	6	7
G0CWS8	60 Kda Chaperonin				11	11	11
G0CWT2	Uncharacterized Protein				1	1	1
G0CWT4	Inosine-5'-Monophosphate Dehydrogenase				1	4	2
G0CWT8	Gmp Synthase [Glutamine-Hydrolyzing]				9	9	9
G0CWU7	Putative Secreted Protein				7	8	9
G0CWU8	Uncharacterized Protein				4	3	4
G0CWV1	Manganese Abc Transporter, Substrate-Binding Protein				3	5	5
G0CWV7	Bifunctional Protein Fold				2	2	1
G0CWW2	Cell-Surface Hemin Receptor				16	14	15
G0CWW3	Iron Abc Transport System Substrate-Binding Protein				5	6	8
G0CWX2	Aminopeptidase N				4	8	6
G0CWX8	Uncharacterized Protein				5	5	12
G0CWZ3	Putative Secreted Protein				1	2	2
G0CWZ4	Putative Secreted Protein				18	20	20
G0CWZ5	Uncharacterized Protein				1	1	1
G0CX18	Uncharacterized Protein				1	2	2
G0CX25	Fatty Acid Synthase				17	20	24
G0CX30	Uncharacterized Protein				17	17	17
G0CX36	Ribonuclease Ph				2	2	3
G0CX49	Isocitrate Dehydrogenase				13	24	22
G0CX54	Venom Serine Protease Kn13				3	5	4
G0CX59	Uncharacterized Protein				2	4	2
G0CX68	Pyruvate Carboxylase				10	17	15
G0CX71	Acyl-Coa Carboxylase Alpha Subunit				15	17	13
G0CX72	Sulfurtransferase				3	4	8
G0CX73	Uncharacterized Protein				6	3	4
G0CX80	Acyl-Coa Carboxylase Complex Subunit				2	4	3
G0CX95	Putative Secreted Protein				12	14	14
G0CX96	Uncharacterized Protein				2	3	2
G0CXA3	Phosphomannomutase				3	7	6
G0CXA5	Mannose-6-Phosphate Isomerase				2	4	5
G0CXA8	Adenosylhomocysteinase				2	5	4
G0CXB2	Lipoprotein Lpqb				7	6	5
G0CXB4	Uncharacterized Protein				2	2	3
G0CXB5	Protein Translocase Subunit Seca				2	4	5
G0CXD0	Putative Secreted Protein				6	6	10
G0CXF9	Cysteine Synthase				12	11	9
G0CXI0	Uncharacterized Protein				2	2	1
G0CXI2	Phosphoribosylformylglycinamidine Cyclo-Ligase				2	3	5

G0CXJ7	Phosphoribosylamine--Glycine Ligase				2	5	4
G0CXK4	Putative Secreted Protein				9	13	13
G0CXL9	Putative Secreted Protein				2	3	2
G0CXM0	Putative Secreted Protein				1	2	1
G0CXM2	Upf0182 Protein Culc22_00609				9	13	12
G0CXM5	Uncharacterized Protein				4	5	6
G0CXN6	Putative Secreted Protein				10	9	10
G0CXN7	Uncharacterized Protein				2	1	2
G0CXN9	Peptide Chain Release Factor 2				1	3	4
G0CXP7	Putative Iron Abc Transport System, Solute-Binding Protein				6	7	5
G0CXR5	Phosphoserine Transaminase				3	3	2
G0CXS5	Putative Secreted Protein				3	3	4
G0CXS7	Uncharacterized Protein				3	1	1
G0CXT3	Abc Transporter Solute-Binding Protein				4	6	4
G0CXU0	Putative Secreted Lpxtg Protein				7	2	3
G0CXU1	Uncharacterized Protein				3	1	4
G0CXU8	Basic 30 Kda Endochitinase				2	5	5
G0CXU9	Putative Adhesin				2	1	1
G0CXV6	Uncharacterized Protein				5	6	5
G0CXV7	Uncharacterized Protein				4	4	3
G0CXW1	Uncharacterized Protein				3	3	4
G0CXW2	Uncharacterized Protein				8	9	14
G0CXW6	Uncharacterized Protein				4	1	1
G0CXX2	Uncharacterized Protein				3	2	2
G0CXX8	Uncharacterized Protein				6	7	6
G0CXY6	Uncharacterized Protein				1	1	2
G0CXZ7	Uncharacterized Protein				1	1	1
G0CY02	Cysteine--Trna Ligase				1	3	5
G0CY07	Putative Secreted Protein				11	11	15
G0CY14	Atp-Dependent Clp Protease Atp-Binding Subunit				7	9	13
G0CY17	Inosine-Uridine Preferring Nucleoside Hydrolase				1	1	2
G0CY21	Laminin Subunit Beta-4				6	4	2
G0CY38	Glucose-6-Phosphate Isomerase				4	5	7
G0CY45	Bifunctional Purine Biosynthesis Protein Purh				2	7	10
G0CY47	Glutamate Abc Transport System Substrate-Binding Protein				6	10	10
G0CY61	Trypsin-Like Serine Protease				6	8	8
G0CY77	Methionine--Trna Ligase				2	5	5
G0CY79	Resuscitation-Promoting Factor				11	14	14
G0CY90	Uncharacterized Protein				2	2	2
G0CYA7	Trypsin-Like Serine Protease				3	9	6
G0CYA8	Lysine--Trna Ligase				2	6	5
G0CYC6	D-Alanyl-D-Alanine Carboxypeptidase				7	4	6
G0CYD0	Putative Secreted Protein				11	14	16

G0CYD3	Uncharacterized Protein				4	2	4
G0CYD5	Uncharacterized Protein				2	3	2
G0CYE0	60 Kda Chaperonin				8	9	14
G0CYE1	Uncharacterized Protein				9	9	7
G0CYE4	Putative Secreted Protein				33	24	27
G0CYF3	Putative Secreted Protein				3	4	2
G0CYG5	Acetate Kinase				10	14	10
G0CYG6	Phosphate Acetyltransferase				7	6	7
G0CYG7	Ferredoxin/Ferredoxin-Nadp Reductase				11	11	13
G0CYH5	Uncharacterized Protein				1	2	3
G0CYH8	Uncharacterized Protein				2	2	2
G0CYI3	Cytochrome C-552				5	5	9
G0CYI9	Putative Secreted Protein				23	27	27
G0CYJ2	Bifunctional Protein Glmu				2	1	1
G0CYK3	Uncharacterized Protein				1	2	2
G0CYL6	Serine Hydroxymethyltransferase				12	9	13
G0CYM0	Putative Secreted Protein				3	3	3
G0CYM5	Fructose-1,6-Bisphosphatase				3	7	6
G0CYM9	4-Hydroxy-3-Methylbut-2-Enyl Diphosphate Reductase				1	1	2
G0CYN2	Ribosome-Binding Atpase Ychf				1	6	6
G0CYN4	Uncharacterized Protein				1	1	2
G0CYP6	Putative Secreted Protein				22	20	25
G0CYQ2	Macrolide Export Atp-Binding/Permease Protein				1	1	2
G0CYQ4	Uncharacterized Protein				1	3	1
G0CYQ6	Putative Secreted Protein				8	9	9
G0CYQ8	Uncharacterized Protein				2	2	2
G0CYR2	N-Succinyldiaminopimelate Aminotransferase				1	1	1
G0CYR6	2,3,4,5-Tetrahydropyridine-2,6-Dicarboxylate N-Succinyltransferase				2	3	2
G0CYS6	Putative Secreted Lpxtg Protein				8	10	10
G0CYS7	Putative Secreted Protein				44	53	53
G0CYT4	Glycerol-3-Phosphate Dehydrogenase				2	3	4
G0CYT6	Surface-Anchored Protein, Fimbrial Subunit				5	8	10
G0CYT9	Surface-Anchored Protein, Fimbrial Subunit				6	8	12
G0CYU1	Chaperone Protein Clpb				2	3	8
G0CYU5	Putative Secreted Protein				2	2	2
G0CYV0	Putative Membrane Protein				2	3	2
G0CYV2	Uncharacterized Protein				17	15	14
G0CYV7	Putative Secreted Protein				1	2	1
G0CYV8	Corynebacterial Protease Cp40				2	5	2
G0CYW3	Surface-Anchored Protein, Fimbrial Subunit				42	44	49
G0CYX0	Uncharacterized Protein				5	14	15

G0CYX8	Abc Transport System, Substrate-Binding Protein				5	5	8
G0CZ02	Uncharacterized Protein				10	16	13
G0CZ10	Uncharacterized Protein				4	3	4
G0CZ21	Putative Secreted Protein				1	2	1
G0CZ55	Arginine--Trna Ligase				5	14	13
G0CZ64	Uncharacterized Protein				2	3	3
G0CZ80	Uncharacterized Protein				2	3	3
G0CZ81	1,4-Alpha-Glucan Branching Enzyme Glgb				7	9	7
G0CZA0	Putative Secreted Protein				4	8	7
G0CZA3	Polyketide Synthase				8	13	11
G0CZA4	Acyl-Coa Synthetase				26	21	23
G0CZA5	Envelope Lipids Regulation Factor				2	5	3
G0CZA7	Trehalose Corynomycolyl Transferase				46	49	46
G0CZB6	Putative Secreted Lpxtg Protein				4	6	4
G0CZB7	Glycerophosphoryl Diester Phosphodiesterase				14	20	20
G0CZC3	Putative Secreted Protein				18	20	20
G0CZD6	Cell-Surface Hemin Receptor				2	4	1
G0CZD8	Sgnh-Hydrolase Family Protein				1	2	4
G0CZD9	Uncharacterized Protein				4	4	5
G0CZE8	Superoxide Dismutase				5	5	4
G0CZE9	Uncharacterized Protein				1	2	2
G0CZG1	Putative Secreted Protein				5	3	3
G0CZH8	Glutamyl-Trna(Gln) Amidotransferase Subunit A				4	4	7
G0CZI4	Putative Secreted Protein				15	11	12
G0CZJ2	Putative Secreted Protein				9	12	11
G0CZJ9	Ketol-Acid Reductoisomerase				6	8	7
G0CZK0	Putative Membrane-Anchored Protein				1	3	4
G0CZK4	D-3-Phosphoglycerate Dehydrogenase				2	4	6
G0CZL2	Glutamate--Trna Ligase				12	9	11
G0CZL3	Uncharacterized Protein				8	11	11
G0CZL4	Putative Secreted Protein				11	16	15
G0CZP7	Pts System Glucose-Specific Component Ii				2	5	3
G0CZR4	Single-Stranded Dna-Binding Protein				1	1	2
G0CZR8	Penicillin-Binding Protein				6	10	8
G0CZS6	Cell-Surface Hemin Receptor				1	4	5
G0CZS7	Dna Protection During Starvation Protein				1	2	1
G0CZT1	Putative Membrane Protein				5	7	5
G0CZT2	Leucine--Trna Ligase				14	16	18
G0CZT5	Putative Secreted Protein				5	5	5
G0CZT8	Htaa Protein				3	4	4
G0CZU1	Putative Secreted Lpxtg Protein				5	6	5
G0CZU2	Glucosamine-6-Phosphate Deaminase				2	2	2

G0CZU5	N-Acetylglucosamine Kinase				1	1	1
G0CZV1	Dihydrodipicolinate Synthase				2	2	6
G0CZV2	Putative Secreted Protein				3	3	4
G0CZV5	4-Aminobutyrate Aminotransferase				2	3	6
G0CZV7	Uncharacterized Protein				2	5	3
G0CZW4	Upf0371 Protein Culc22_02319				2	3	6
G0CZY6	Putative Secreted Protein				2	2	7
G0CZY9	Thioredoxin Reductase				2	4	3

Supplementary table 2. List of proteins identified as cell surface proteins of *C. ulcerans* 809 and BR-AD22. Accession numbers, description and identified peptides per sample.

Secretome proteins		*Corynebacterium ulcerans* 809			*Corynebacterium ulcerans* BR-AD22		
Accession	Description	Peptides 1	Peptides 2	Peptides 3	Peptides 1	Peptides 2	Peptides 3
embl-cds:AEG80555	Putative Secreted Protein	1	1	2			
embl-cds:AEG80558	Hypothetical Protein	5	1	2			
embl-cds:AEG80559	Peptidyl-Prolyl Cis-Trans Isomerase	3	1	1			
embl-cds:AEG80585	Iron-Siderophore Binding Protein	3	2	6			
embl-cds:AEG80589	Penicillin-Binding Protein	4	4	3			
embl-cds:AEG80616	Anti-Sigma Factor	1	2	2			
embl-cds:AEG80629	Putative Secreted Protein	7	7	6			
embl-cds:AEG80637	Putative Secreted Protein	8	6	5			
embl-cds:AEG80639	1,4-Alpha-Glucan-Branching Enzyme	3	5	9			
embl-cds:AEG80657	Hypothetical Protein	3	1	3			
embl-cds:AEG80681	Glycerol Kinase	4	1	1			
embl-cds:AEG80685	Hypothetical Protein	2	2	2			
embl-cds:AEG80713	Hypothetical Protein	1	2	2			
embl-cds:AEG80719	Putative Secreted Protein	2	1	3			
embl-cds:AEG80744	Hypothetical Protein	1	1	1			
embl-cds:AEG80748	Laminin Subunit Beta-1	4	5	5			
embl-cds:AEG80754	Deoxyribose-Phosphate Aldolase	1	2	3			
embl-cds:AEG80756	Putative Secreted Lpxtg Protein	4	3	4			
embl-cds:AEG80757	Putative Secreted Protein	22	24	33			
embl-cds:AEG80768	Putative Secreted Protein	1	3	2			

embl-cds:AEG80769	Laminin Subunit Beta-2	10	10	15			
embl-cds:AEG80780	Putative Membrane Protein	5	5	6			
embl-cds:AEG80781	Putative Secreted Protein	3	2	2			
embl-cds:AEG80785	Putative Secreted Protein	9	7	11			
embl-cds:AEG80821	Putative Secreted Protein	8	6	6			
embl-cds:AEG80850	Putative Secreted Protein	6	4	4			
embl-cds:AEG80904	Putative Secreted Protein	1	1	2			
embl-cds:AEG80905	Putative Secreted Protein	3	3	4			
embl-cds:AEG80907	Dna-Directed Rna Polymerase Beta' Subunit	3	4	5			
embl-cds:AEG80909	Putative Secreted Lpxtg Protein	2	2	2			
embl-cds:AEG80955	Non-Specific Acid Phosphatase	4	6	8			
embl-cds:AEG80957	50S Ribosomal Protein L6	2	2	3			
embl-cds:AEG80969	Hypothetical Protein	1	2	2			
embl-cds:AEG80972	Adenylate Kinase	7	7	6			
embl-cds:AEG80974	Sialidase Precursor	16	12	12			
embl-cds:AEG80975	Putative Secreted Protein	3	2	2			
embl-cds:AEG81006	Molecular Chaperone	4	4	4			
embl-cds:AEG81014	Gmp Synthase	1	1	1			
embl-cds:AEG81023	Putative Secreted Protein	2	2	2			
embl-cds:AEG81038	Cell-Surface Hemin Receptor	2	1	2			
embl-cds:AEG81044	Isocitrate Dehydrogenase	14	9	9			
embl-cds:AEG81049	Venom Serine Protease Kn13	1	1	1			
embl-cds:AEG81065	Acyl-Coa Carboxylase Alpha Subunit	6	5	7			
embl-cds:AEG81067	Hypothetical Protein	6	4	6			
embl-cds:AEG81071	Ribokinase	1	1	1			
embl-cds:AEG81085	Putative Iron Compound Abc Transporter Iron Compound Binding Protein	1	1	2			
embl-cds:AEG81091	Putative Secreted Protein	9	7	7			
embl-cds:AEG81099	Phosphomannomutase	3	2	1			
embl-cds:AEG81107	Putative Secreted Protein	2	1	1			

embl-cds:AEG81115	Hypothetical Protein	2	1	4			
embl-cds:AEG81125	Putative Secreted Protein	1	2	4			
embl-cds:AEG81137	Hypothetical Protein	1	1	2			
embl-cds:AEG81138	Putative Secreted Protein	1	1	1			
embl-cds:AEG81141	Putative Membrane Protein	6	3	7			
embl-cds:AEG81154	Putative Secreted Protein	3	1	3			
embl-cds:AEG81165	Ferric Anguibactin-Binding Protein	1	1	1			
embl-cds:AEG81227	Glutamate Abc Transport System Substrate-Binding Protein	1	1	1			
embl-cds:AEG81241	Trypsin-Like Serine Protease	5	4	5			
embl-cds:AEG81243	Hypothetical Protein	1	1	1			
embl-cds:AEG81259	Resuscitation-Promoting Factor	2	3	3			
embl-cds:AEG81272	Glyceraldehyde 3-Phosphate Dehydrogenase	5	3	3			
embl-cds:AEG81291	Putative Secreted Protein	2	1	1			
embl-cds:AEG81313	Putative Secreted Protein	1	1	1			
embl-cds:AEG81322	4-Hydroxy-3-Methylbut-2-Enyl Diphosphate Reductase	2	1	1			
embl-cds:AEG81325	Hypothetical Protein	5	3	3			
embl-cds:AEG81338	Putative Secreted Protein	5	4	7			
embl-cds:AEG81344	Macrolide Export Atp-Binding/Permease Protein	1	4	3			
embl-cds:AEG81348	Putative Secreted Protein	3	2	3			
embl-cds:AEG81358	2,3,4,5-Tetrahydropyridine-2-Carboxylate N-Succinyltransferase	4	2	2			
embl-cds:AEG81379	Putative Secreted Protein	3	2	4			
embl-cds:AEG81413	Arginyl-Trna Synthetase	7	4	3			
embl-cds:AEG81419	Transcription Termination Factor	1	1	3			
embl-cds:AEG81445	Electron Transfer Flavoprotein Alpha Subunit	2	1	1			
embl-cds:AEG81460	Putative Secreted Protein	4	3	5			
embl-cds:AEG81469	Putative Membrane Protein	1	1	1			
embl-cds:AEG81476	Putative Membrane-Anchored Protein	4	4	3			
embl-cds:AEG81488	Glutamyl-Trna Synthetase	3	1	1			
embl-cds:AEG81505	D-Alanine-D-Alanine Ligase	2	1	1			

embl-cds:AEG81556	Putative Secreted Protein	8	6	8			
embl-cds:AEG81605	Putative Secreted Protein	3	2	1			
embl-cds:AEG81645	Putative Secreted Protein	4	4	4			
embl-cds:AEG81666	Resuscitation-Promoting Factor Interacting Protein	7	8	6			
embl-cds:AEG81667	Hypothetical Protein	1	3	3			
embl-cds:AEG81668	Aconitase	6	4	4			
embl-cds:AEG81681	Chromosome-Associated Kinesin Kif4A	1	2	2			
embl-cds:AEG81688	Hypothetical Protein	1	1	1			
embl-cds:AEG81733	Transketolase	3	2	2			
embl-cds:AEG81734	Transaldolase	3	1	3			
embl-cds:AEG81740	Triosephosphate Isomerase	2	5	5			
embl-cds:AEG81741	Phosphoglycerate Kinase	8	4	6			
embl-cds:AEG81742	Glyceraldehyde 3-Phosphate Dehydrogenase	3	2	4			
embl-cds:AEG81788	Putative Membrane Protein	1	1	2			
embl-cds:AEG81792	Histidyl-Trna Synthetase	1	1	1			
embl-cds:AEG81794	Thiol Peroxidase	1	1	3			
embl-cds:AEG81795	Peptidyl-Prolyl Cis-Trans Isomerase	1	1	1			
embl-cds:AEG81836	Rna Polymerase Sigma Factor A	1	1	1			
embl-cds:AEG81910	Putative Secreted Protein	4	3	3			
embl-cds:AEG81927	Penicillin-Binding Protein	5	6	5			
embl-cds:AEG81966	Signal Recognition Particle Receptor	1	1	1			
embl-cds:AEG82019	Isoleucyl-Trna Synthetase	4	4	2			
embl-cds:AEG82025	Cell Division Protein	5	6	5			
embl-cds:AEG82027	Udp-N-Acetylmuramate--Alanine Ligase	2	1	2			
embl-cds:AEG82033	Udp-N-Acetylmuramoylalanyl-D-Glutamate--2,6-Diaminopimelate Ligase	1	1	1			
embl-cds:AEG82034	Penicillin-Binding Protein	5	5	5			
embl-cds:AEG82047	Phospho-2-Dehydro-3-Deoxyheptonate Aldolase	2	1	1			
embl-cds:AEG82070	Dihydrolipoamide Acyltransferase	1	1	1			
embl-cds:AEG82072	Glycine Cleavage System T Protein	4	4	3			

embl-cds:AEG82080	Iron-Siderophore Binding Protein	2	1	2			
embl-cds:AEG82093	Threonine Synthase	4	4	3			
embl-cds:AEG82097	Heme Oxygenase	3	3	2			
embl-cds:AEG82103	Galactokinase	1	1	1			
embl-cds:AEG82132	Glycyl-Trna Synthetase	4	1	1			
embl-cds:AEG82157	Putative Secreted Protein	2	1	2			
embl-cds:AEG82165	Putative Secreted Protein	5	7	5			
embl-cds:AEG82190	Hypothetical Protein	2	1	2			
embl-cds:AEG82205	Dihydrofolate Synthase / Folylpolyglutamate Synthase	1	1	1			
embl-cds:AEG82213	Trigger Factor	3	3	4			
embl-cds:AEG82238	Putative Secreted Protein	4	4	5			
embl-cds:AEG82239	Putative Secreted Protein	6	4	6			
embl-cds:AEG82241	Corynebacterineae Mycolate Reductase A	1	1	1			
embl-cds:AEG82244	Hypothetical Protein	3	2	4			
embl-cds:AEG82252	Fatty Acid Synthase	8	7	10			
embl-cds:AEG82258	Hypothetical Protein	2	1	1			
embl-cds:AEG82283	Protein Piccolo	1	1	1			
embl-cds:AEG82284	Hypothetical Protein	5	4	4			
embl-cds:AEG82285	Putative Secreted Protein	5	4	5			
embl-cds:AEG82316	Putative Secreted Protein	2	1	2			
embl-cds:AEG82337	Phosphoribosylamine-Glycine Ligase	8	6	3			
embl-cds:AEG82344	Putative Secreted Protein	7	7	8			
embl-cds:AEG82363	Putative Secreted Protein	3	4	3			
embl-cds:AEG82375	Laminin Subunit Alpha-1	11	10	10			
embl-cds:AEG82395	D-Alanyl-D-Alanine Carboxypeptidase	7	5	8			
embl-cds:AEG82397	Hypothetical Protein	1	1	1			
embl-cds:AEG82399	Putative Secreted Protein	2	2	2			
embl-cds:AEG82407	Hypothetical Protein	2	2	2			
embl-cds:AEG82409	Hypothetical Protein	2	2	2			
embl-cds:AEG82410	Hypothetical Protein	2	2	2			

embl-cds:AEG82411	Molecular Chaperone	5	2	4			
embl-cds:AEG82436	Acetate Kinase	6	3	5			
embl-cds:AEG82437	Phosphate Acetyltransferase	5	5	4			
embl-cds:AEG82438	Ferredoxin/Ferredoxin-Nadp Reductase	7	7	6			
embl-cds:AEG82447	Hypothetical Protein	3	3	3			
embl-cds:AEG82448	Hypothetical Protein	2	2	2			
embl-cds:AEG82449	Hypothetical Protein	4	4	4			
embl-cds:AEG82454	Putative Secreted Protein	2	1	2			
embl-cds:AEG82460	Putative Secreted Protein	12	10	13			
embl-cds:AEG82467	Putative Secreted Protein	41	41	44			
embl-cds:AEG82474	Glycerol-3-Phosphate Dehydrogenase	3	1	3			
embl-cds:AEG82476	Surface-Anchored Protein, Fimbrial Subunit	2	1	1			
embl-cds:AEG82484	Laminin Subunit Alpha-2	7	7	8			
embl-cds:AEG82485	Putative Secreted Protein	7	5	6			
embl-cds:AEG82495	Hypothetical Protein	14	14	16			
embl-cds:AEG82506	Surface-Anchored Protein, Fimbrial Subunit	5	6	6			
embl-cds:AEG82513	Hypothetical Protein	1	1	1			
embl-cds:AEG82521	Abc Transport System, Substrate-Binding Protein	3	4	4			
embl-cds:AEG82537	Hypothetical Protein	2	1	2			
embl-cds:AEG82544	Hypothetical Protein	10	7	9			
embl-cds:AEG82562	Acyl-Coa Synthetase	17	14	15			
embl-cds:AEG82563	Envelope Lipids Regulation Factor	2	2	2			
embl-cds:AEG82565	Trehalose Corynomycolyl Transferase	10	5	11			
embl-cds:AEG82571	Glycosyltransferase	1	1	1			
embl-cds:AEG82574	Glycerophosphoryl Diester Phosphodiesterase	8	7	7			
embl-cds:AEG82580	Putative Secreted Protein	10	6	9			
embl-cds:AEG82593	Cell-Surface Hemin Receptor	4	3	5			
embl-cds:AEG82616	Housekeeping Sortase	2	2	1			
embl-cds:AEG82641	Penicillin-Binding Protein	10	10	9			
embl-cds:AEG82649	Cell-Surface Hemin Receptor	3	1	3			

embl-cds:AEG82655	Leucyl-Trna Synthetase	3	2	3			
embl-cds:AEG82657	Putative Membrane Protein	1	1	1			
G0CTA7;embl-cds:AEG81533	Translation Initiation Factor If-3		2	2	1	1	1
G0CTA8;embl-cds:AEG81534	50S Ribosomal Protein L35	1			1	1	1
G0CTA9;embl-cds:AEG81535	50S Ribosomal Protein L20	1	1	2	3	3	2
G0CTB3;embl-cds:AEG81538	Phenylalanine--Trna Ligase Alpha Subunit	1			6	3	1
G0CTC1;embl-cds:AEG81546	Argininosuccinate Synthase	2	1	1	6	5	7
G0CTC3;embl-cds:AEG81548	Uncharacterized Protein		1	1	1	1	1
G0CTC4;embl-cds:AEG81549	Tyrosine--Trna Ligase	1		1	3	2	1
G0CTE3;embl-cds:AEG81568	Gtpase Der	1		1	5	1	2
G0CTF6;embl-cds:AEG81581	Uncharacterized Protein			1	1	1	1
G0CTF8;embl-cds:AEG81583	Inhibitor Of Odh Activity	1	1	1	2	1	3
G0CTI2;embl-cds:AEG81607	Rna Polymerase-Binding Protein Rbpa				2	1	1
G0CTI3;embl-cds:AEG81608	Polyprenol-Phosphate-Mannose Synthase Domain 1				1	1	1
G0CTI8;embl-cds:AEG82714	Putative Hydrolase	1			2	3	3
G0CTN6;embl-cds:AEG81651	Dihydroxyacetone Kinase	2	2	1	1	1	1
G0CTP2;embl-cds:AEG81657	Uncharacterized Protein				3	3	3
G0CTP6;embl-cds:AEG81661	Putative Secreted Protein	1		2	7	5	7
G0CTQ6;embl-cds:AEG81671	Upf0237 Protein Culc22_01153				1	1	1
G0CTU9;embl-cds:AEG81716	Uncharacterized Protein				3	1	1
G0CTV7;embl-cds:AEG81724	Abc-Type Transport System Involved In Fe-S Cluster Assembly Atp-Binding Protein	3	1	2	8	3	4
G0CTX8;embl-cds:AEG81745	Nucleotide-Binding Protein Culc22_01225				3	1	1
G0CTZ3;embl-cds:AEG81759	Dna-Directed Rna Polymerase Subunit Omega				1	1	1
G0CTZ5;embl-cds:AEG81761	Corynebacterial Integration Host Factor	2	2	2	4	3	3
G0CU06;embl-cds:AEG81772	Elongation Factor Ef-P	2	2	1	3	2	2
G0CU14;embl-cds:AEG81780	Putative Secreted Protein	3	4	4	3	3	3

G0CU19;embl-cds:AEG80547	Dna Gyrase Subunit B			1	8	4	2
G0CU20;embl-cds:AEG80548	Uncharacterized Protein	2	2	2	6	2	2
G0CU23;embl-cds:AEG80551	Dna Gyrase Subunit A			2	7	1	4
G0CU44;embl-cds:AEG81799	Putative Secreted Protein	2	1	1	2	1	2
G0CU45;embl-cds:AEG81800	Protein-Export Membrane Protein Secf	1		1	7	4	2
G0CU51;embl-cds:AEG81806	Probable Transcriptional Regulatory Protein Culc22_01288	1	2	1	3	3	
G0CU61;embl-cds:AEG81816	Threonine--Trna Ligase				4	2	3
G0CU89;embl-cds:AEG81844	Dtxr-Family Transcription Regulator	6	2	3	6	6	7
G0CU95;embl-cds:AEG81850	Alkyl Hydroperoxide Reductase	2	2	1	3	3	3
G0CUA2;embl-cds:AEG81857	Deor-Family Transcription Regulator				1	2	1
G0CUC6;embl-cds:AEG80578	Uncharacterized Protein	2	1	1	4	2	2
G0CUE0;embl-cds:AEG80592	Putative Secreted Protein				1	2	2
G0CUG4;embl-cds:AEG80613	Uncharacterized Protein	1	1	1	1	1	1
G0CUH6;embl-cds:AEG80631	Uncharacterized Protein	9	9	10	9	8	6
G0CUI8;embl-cds:AEG81863	Phosphocarrier Protein Hpr	3	3	2		2	1
G0CUK4;embl-cds:AEG81879	Protein Reca		1	1	6	5	4
G0CUK9;embl-cds:AEG81884	Uncharacterized Protein	5	2	5	10	5	8
G0CUM1;embl-cds:AEG81896	Polyribonucleotide Nucleotidyltransferase	1	1	2	15	6	8
G0CUM2;embl-cds:AEG81897	30S Ribosomal Protein S15	2	1	1	3	3	2
G0CUN0;embl-cds:AEG81905	Ribosome-Binding Factor A	2	1	2	2	1	2
G0CUN1;embl-cds:AEG81906	Translation Initiation Factor If-2	2	2	3	16	11	10
G0CUN3;embl-cds:AEG81908	Transcription Termination/Antitermination Protein Nusa				2	3	3
G0CUP3;embl-cds:AEG81918	Cobalamin Adenosyltransferase	3			2	2	4

G0CUQ4;embl-cds:AEG81928	4-Hydroxy-3-Methylbut-2-En-1-Yl Diphosphate Synthase (Flavodoxin)				5	2	2
G0CUQ7;embl-cds:AEG81931	Uncharacterized Protein			1	3	2	2
G0CUQ8;embl-cds:AEG81932	Probable Dual-Specificity Rna Methyltransferase Rlmn				2	2	1
G0CUR1;embl-cds:AEG81935	Ribosome-Recycling Factor	3	3	3	4	5	4
G0CUR2;embl-cds:AEG81936	Uridylate Kinase	1			5	5	4
G0CUR3;embl-cds:AEG81937	Elongation Factor Ts	5	2	4	11	3	5
G0CUR4;embl-cds:AEG81938	30S Ribosomal Protein S2	4	2	3	9	5	7
G0CUV8;embl-cds:AEG80678	Uncharacterized Protein				1	1	1
G0CUW8;embl-cds:AEG80687	Putative Secreted Protein	7	5	4	7	3	6
G0CUX1;embl-cds:AEG80690	Decaprenylphosphoryl-Beta-D-Ribose 2-Epimerase Component	1	1	1	5	4	3
G0CUY4;embl-cds:AEG80703	Uncharacterized Protein				1	1	2
G0CV12;embl-cds:AEG81955	50S Ribosomal Protein L19	2	1	1	4	2	3
G0CV16;embl-cds:AEG81959	Trna (Guanine-N(1)-)-Methyltransferase	1	1	1	1	2	2
G0CV31;embl-cds:AEG81974	Uncharacterized Protein		2		6	7	5
G0CV41;embl-cds:AEG81983	Putative Secreted Protein	2	2	2	4	2	1
G0CV49;embl-cds:AEG81991	1-(5-Phosphoribosyl)-5-[(5-Phosphoribosylamino)Methylideneamino] Imidazole-4-Carboxamide Isomerase	5	2	1		3	5
G0CV58;embl-cds:AEG82000	Putative Secreted Protein	2	1	3	5	2	1
G0CV68;embl-cds:AEG82010	Dna-Directed Dna Polymerase		1	1	1	1	1
G0CV74;embl-cds:AEG82016	Putative Secreted Protein	1		1	1	1	1
G0CV79;embl-cds:AEG82021	Uncharacterized Protein	4	2	3	8	7	3
G0CV86;embl-cds:AEG80721	Pyridoxal 5'-Phosphate Synthase Subunit Pdxs	1	1	2	5	3	6
G0CV98;embl-cds:AEG80733	Uncharacterized Protein	4	1	1		3	4
G0CVA1;embl-cds:AEG80736	Nucleoid-Associated Protein Culc22_00193	2	1		1	1	1

G0CVB1;embl-cds:AEG80746	Uncharacterized Protein				3	1	1
G0CVC7;embl-cds:AEG80762	Aspartokinase		2	2	5	1	4
G0CVC9;embl-cds:AEG80764	Aspartate-Semialdehyde Dehydrogenase	2	2	2	2	4	3
G0CVF9;embl-cds:AEG80790	Crp-Family Transcription Regulator	1		3	7	4	2
G0CVG6;embl-cds:AEG80797	Uncharacterized Protein			1	2	2	1
G0CVH1;embl-cds:AEG82030	Udp-N-Acetylmuramoylalanine--D-Glutamate Ligase	3	2		2	3	2
G0CVH7;embl-cds:AEG82036	Ribosomal Rna Small Subunit Methyltransferase H	2	2	2	3	2	2
G0CVI6;embl-cds:AEG82045	Uncharacterized Protein				1	1	2
G0CVJ1;embl-cds:AEG82050	1-Acyl-Sn-Glycerol-3-Phosphate Acyltransferase				4	1	1
G0CVJ6;embl-cds:AEG82055	Ubiquinol-Cytochrome C Reductase Cytochrome B Subunit	1			6	2	2
G0CVJ7;embl-cds:AEG82056	Ubiquinol-Cytochrome C Reductase Iron-Sulfur Subunit		1	1	3	1	3
G0CVK3;embl-cds:AEG82062	Uncharacterized Protein				2	1	1
G0CVK8;embl-cds:AEG82067	Branched-Chain-Amino-Acid Aminotransferase	4	3	4	1	4	5
G0CVL7;embl-cds:AEG82076	Lipoyl Synthase				4	2	3
G0CVM9;embl-cds:AEG82088	Uncharacterized Protein				8	4	3
G0CVN0;embl-cds:AEG82089	Uncharacterized Protein	2		1	3	2	1
G0CVR2;embl-cds:AEG80808	Cold Shock-Like Protein A	2		3	3	2	2
G0CVS1;embl-cds:AEG80815	Putative Secreted Protein	5	4	4	6	8	7
G0CVT0;embl-cds:AEG80823	Dihydrolipoyl Dehydrogenase	1	1		2	2	2
G0CVT3;embl-cds:AEG80826	Succinate Dehydrogenase Flavoprotein Subunit	1	1	1	7	1	2
G0CVT4;embl-cds:AEG80827	Succinate Dehydrogenase Iron-Sulfur Protein	1	1		2	1	1

G0CVT9;embl-cds:AEG80832	Putative Secreted Protein	2	3	2	4	3	3
G0CVU1;embl-cds:AEG80834	Formate Acetyltransferase	1	1	1	2	2	1
G0CVU2;embl-cds:AEG80835	Formate Acetyltransferase	1		1	7	4	2
G0CVU4;embl-cds:AEG80837	Uncharacterized Protein	1	1	1	4	1	2
G0CVU8;embl-cds:AEG80841	2,3-Bisphosphoglycerate-Dependent Phosphoglycerate Mutase	4	1	3	10	4	5
G0CVX0;embl-cds:AEG80863	Uncharacterized Protein	3	1	1	4	2	2
G0CW00;embl-cds:AEG82124	Glutamine--Fructose-6-Phosphate Aminotransferase [Isomerizing]	1	1	1	5	1	1
G0CW10;embl-cds:AEG82134	Fur-Family Transcription Regulator				4	3	3
G0CW24;embl-cds:AEG82148	Uncharacterized Protein	2	1	2	11	5	2
G0CW25;embl-cds:AEG82149	4-Alpha-Glucanotransferase				2	1	2
G0CW81;embl-cds:AEG80892	50S Ribosomal Protein L11		1	2	3	2	2
G0CW82;embl-cds:AEG80893	50S Ribosomal Protein L1	5	2	3	9	7	7
G0CW86;embl-cds:AEG80897	50S Ribosomal Protein L10				5	2	1
G0CW87;embl-cds:AEG80898	50S Ribosomal Protein L7/L12	6	5	6	10	7	7
G0CW95;embl-cds:AEG80906	Dna-Directed Rna Polymerase Subunit Beta	1		1	16	4	5
G0CWB0;embl-cds:AEG80921	Uncharacterized Protein	1	1	1	2	2	3
G0CWB2;embl-cds:AEG80923	30S Ribosomal Protein S7	3	4	4	7	5	5
G0CWB3;embl-cds:AEG80924	Elongation Factor G	4	2	3	23	10	9
G0CWB4;embl-cds:AEG80925	Elongation Factor Tu	9	9	6	15	9	10
G0CWC3;embl-cds:AEG80934	30S Ribosomal Protein S10	4	3	3	7	5	5

G0CWC4;embl-cds:AEG80935	50S Ribosomal Protein L3	3	2	1	6	3	4
G0CWC5;embl-cds:AEG80936	50S Ribosomal Protein L4	3	4	5	7	5	5
G0CWC6;embl-cds:AEG80937	50S Ribosomal Protein L23	2	1	1	4	3	3
G0CWC7;embl-cds:AEG80938	50S Ribosomal Protein L2	5	6	8	12	10	10
G0CWC8;embl-cds:AEG80939	30S Ribosomal Protein S19	3	3	3	5	5	5
G0CWC9;embl-cds:AEG80940	50S Ribosomal Protein L22	3	6	6	5	6	6
G0CWD0;embl-cds:AEG80941	30S Ribosomal Protein S3	3	3	6	9	5	6
G0CWD1;embl-cds:AEG80942	50S Ribosomal Protein L16			2	3	2	2
G0CWD2;embl-cds:AEG80943	50S Ribosomal Protein L29		1		4	4	5
G0CWD3;embl-cds:AEG80944	30S Ribosomal Protein S17	1	2	3	5	3	3
G0CWD4;embl-cds:AEG80945	Putative Secreted Protein				3	6	8
G0CWD8;embl-cds:AEG80949	50S Ribosomal Protein L14	2	1	2	4	2	3
G0CWD9;embl-cds:AEG80950	50S Ribosomal Protein L24				6	3	2
G0CWE0;embl-cds:AEG80951	50S Ribosomal Protein L5	4	2	3	7	7	5
G0CWE5;embl-cds:AEG80956	30S Ribosomal Protein S8	2	1	1	7	3	4
G0CWE7;embl-cds:AEG80958	50S Ribosomal Protein L18	2	2	1	1	2	2
G0CWE8;embl-cds:AEG80959	30S Ribosomal Protein S5	2	2	2	6	3	3
G0CWJ9;embl-cds:AEG82182	30S Ribosomal Protein S20	1	1	1	2	1	1
G0CWL5;embl-cds:AEG82198	50S Ribosomal Protein L27	2	2	3	5	4	2
G0CWL6;embl-cds:AEG82199	50S Ribosomal Protein L21	3	2	3	3	3	4

G0CWM4;embl-cds:AEG82207	Malate Dehydrogenase	5	2	3	5	6	4
G0CWM9;embl-cds:AEG82211	Atp-Dependent Clp Protease Proteolytic Subunit	1	1	1	1	1	1
G0CWN0;embl-cds:AEG82212	Atp-Dependent Clp Protease Proteolytic Subunit				1	1	1
G0CWN2;embl-cds:AEG80960	50S Ribosomal Protein L30	1	1	2	5	3	4
G0CWN3;embl-cds:AEG80961	50S Ribosomal Protein L15	2	4	2	6	2	4
G0CWN5;embl-cds:AEG80963	Maltotriose-Binding Protein	5	5	6	6	3	6
G0CWP8;embl-cds:AEG80977	30S Ribosomal Protein S13	2	2	3	3	3	2
G0CWP9;embl-cds:AEG80978	30S Ribosomal Protein S11				4	1	2
G0CWQ0;embl-cds:AEG80979	30S Ribosomal Protein S4		3	3	12	5	7
G0CWQ1;embl-cds:AEG80980	Dna-Directed Rna Polymerase Subunit Alpha	1	2	3	4	1	2
G0CWQ2;embl-cds:AEG80981	50S Ribosomal Protein L17	2	3	3	7	4	5
G0CWR3;embl-cds:AEG80991	50S Ribosomal Protein L13	2	2	3	3	3	3
G0CWR4;embl-cds:AEG80992	30S Ribosomal Protein S9	2	2	2	6	6	2
G0CWR5;embl-cds:AEG80993	Phosphoglucosamine Mutase				3	1	1
G0CWR9;embl-cds:AEG80997	Uncharacterized Protein				3	3	3
G0CWS7;embl-cds:AEG81005	10 Kda Chaperonin	2	1	2	4	4	4
G0CWT5;embl-cds:AEG81012	Uncharacterized Protein	2			5	6	6
G0CWU3;embl-cds:AEG81019	Putative Secreted Protein	2	3	3	3		1
G0CWW8;embl-cds:AEG82215	Uncharacterized Protein				2	2	2
G0CWX0;embl-cds:AEG82217	Ribose 5-Phosphate Isomerase	2	2	2	4	1	4

G0CWZ7;embl-cds:AEG82242	Oligoribonuclease	2	1	1	3	1	2
G0CX20;embl-cds:AEG82247	Uncharacterized Protein	2	2		2	2	2
G0CX21;embl-cds:AEG82248	Uncharacterized Protein	1			3	3	3
G0CX22;embl-cds:AEG82249	Bacterioferritin Comigratory Protein	2	2	1	1	2	2
G0CX40;embl-cds:AEG82263	Uncharacterized Protein				4	2	1
G0CX62;embl-cds:AEG81057	Uracil Phosphoribosyltransferase	2		1	2	2	2
G0CX66;embl-cds:AEG81061	Dihydrolipoamide Dehydrogenase				5	3	5
G0CX81;embl-cds:AEG81075	Acyl-Coa Carboxylase Complex Subunit	1		1	8	4	4
G0CX97;embl-cds:AEG81093	Lytr-Family Transcription Regulator	3	5	4	2	3	2
G0CX99;embl-cds:AEG81095	Mannose-1-Phosphate Guanyltransferase			1	1	3	3
G0CXB7;embl-cds:AEG81112	Uncharacterized Protein	2	2	2	3	1	2
G0CXB9;embl-cds:AEG81114	Uncharacterized Protein			1	5	3	3
G0CXC9;embl-cds:AEG81124	Putative Secreted Protein	2	4	3	2	3	2
G0CXD1;embl-cds:AEG82272	Cytochrome C Oxidase Subunit I			1	4	1	2
G0CXD5;embl-cds:AEG82276	Ribonucleoside-Diphosphate Reductase				9	3	3
G0CXF7;embl-cds:AEG82297	Luxr-Family Transcription Regulator	1		1	4	3	3
G0CXG1;embl-cds:AEG82301	Uncharacterized Protein	1	1	1		1	1
G0CXG7;embl-cds:AEG82307	Succinyl-Coa:Coenzyme A Transferase	3	1	2	6	3	3
G0CXH8;embl-cds:AEG82318	Upf0678 Fatty Acid-Binding Protein-Like Protein Culc22_01893			1	1	2	1
G0CXI9;embl-cds:AEG82329	Phosphoribosylformylglycinamidine Synthase Subunit Purq				3	1	3
G0CXJ0;embl-cds:AEG82330	Phosphoribosylformylglycinamidine Synthase Subunit Purs	1	1	2	4	2	2
G0CXJ8;embl-cds:AEG82338	Uncharacterized Protein	1		1	1	1	1
G0CXK1;embl-cds:AEG82341	Two-Component System Transcriptional Regulatory Protein	1			1	1	1
G0CXK8;embl-cds:AEG81127	Uncharacterized Protein			1	2	2	2
G0CXM1;embl-cds:AEG81140	Uncharacterized Protein	1		1	1	1	1

G0CXN2;embl-cds:AEG81150	Oxaloacetate Decarboxylase Alpha Subunit				4	1	2
G0CXN3;embl-cds:AEG81151	Methylmalonyl-Coa Decarboxylase Alpha Subunit	1			5	4	4
G0CXP3;embl-cds:AEG81161	Cell Division Protein Ftsx	1		1	7	2	3
G0CXQ2;embl-cds:AEG81170	Uncharacterized Protein				2	2	2
G0CXQ6;embl-cds:AEG81174	Resuscitation-Promoting Factor	4	2	3	4	3	4
G0CXQ9;embl-cds:AEG81178	Putative Secreted Protein	1	2	1	1	1	
G0CXR3;embl-cds:AEG81182	Uncharacterized Protein				1	1	2
G0CXR6;embl-cds:AEG81185	Citrate Synthase	3	2	1	8	6	6
G0CXR9;embl-cds:AEG81188	Superoxide Dismutase [Cu-Zn]	1	3		1	2	2
G0CXS3;embl-cds:AEG81192	Putative Transport Membrane Protein			1	3	2	1
G0CY05;embl-cds:AEG82361	Putative Transcription Factor				3	1	3
G0CY06;embl-cds:AEG82362	Putative Secreted Protein	1	1	3	2	3	2
G0CY28;embl-cds:AEG81208	Uncharacterized Protein	2	3	3	1	2	1
G0CY30;embl-cds:AEG81210	Uncharacterized Protein		1	2	4	1	2
G0CY32;embl-cds:AEG81212	Thymidylate Synthase	2			2	1	2
G0CY53;embl-cds:AEG81233	30S Ribosomal Protein S18	1	1		1	1	1
G0CY55;embl-cds:AEG81235	50S Ribosomal Protein L33			1	1	1	1
G0CY56;embl-cds:AEG81236	50S Ribosomal Protein L28	3	1	2	4	3	3
G0CY58;embl-cds:AEG81238	50S Ribosomal Protein L32	2	2	2	2	2	2
G0CY59;embl-cds:AEG81239	Two-Component System Transcriptional Regulatory Protein	1	1	1	3		4
G0CY64;embl-cds:AEG81244	Large-Conductance Mechanosensitive Channel				2	2	1
G0CY67;embl-cds:AEG81247	Utp--Glucose-1-Phosphate Uridylyltransferase	1	1	1	3	1	1
G0CY87;embl-cds:AEG81267	Uncharacterized Protein				3	1	1
G0CY91;embl-cds:AEG81271	Uncharacterized Protein				1	1	1
G0CY97;embl-cds:AEG81276	Peptide Chain Release Factor 3	1			5	2	1

G0CYA4;embl-cds:AEG81281	50S Ribosomal Protein L25	1	1	1	5	1	1
G0CYB4;embl-cds:AEG82383	Choline Dehydrogenase				4	2	3
G0CYC3;embl-cds:AEG82392	Atp-Dependent Zinc Metalloprotease Ftsh	1	1	1	4	2	4
G0CYC4;embl-cds:AEG82393	Hypoxanthine Phosphoribosyltransferase				1	1	1
G0CYH1;embl-cds:AEG82442	Purine Phosphoribosyltransferase	3	4	4	4	4	4
G0CYH2;embl-cds:AEG82443	Putative Membrane Protein	11	10	12	10	13	12
G0CYJ1;embl-cds:AEG81283	Ribose-Phosphate Pyrophosphokinase				3	1	2
G0CYK0;embl-cds:AEG81292	Enolase	1		1	8	5	7
G0CYK2;embl-cds:AEG81294	Uncharacterized Protein	3	1	2	3	1	3
G0CYK9;embl-cds:AEG81301	Transcription Elongation Factor Grea	2	1	2	3	4	3
G0CYM4;embl-cds:AEG81317	Fumarate Hydratase Class Ii	5	2	6	12	7	12
G0CYP9;embl-cds:AEG81341	Uncharacterized Protein				11	5	5
G0CYQ3;embl-cds:AEG81345	Putative Secreted Protein	1			1	1	1
G0CYQ5;embl-cds:AEG81347	Uncharacterized Protein	2	1	2	1	1	
G0CYQ7;embl-cds:AEG81349	Uncharacterized Protein		2	2	12	7	9
G0CYS8;embl-cds:AEG82468	Fructose-Bisphosphate Aldolase	1	1	2	6	1	2
G0CYT3;embl-cds:AEG82473	Thiosulfate Sulfurtransferase				2	1	4
G0CYV5;embl-cds:AEG82498	Alcohol Dehydrogenase	1	2	1	2	2	1
G0CYV6;embl-cds:AEG82499	Aldehyde Dehydrogenase	3	2	1	4	3	3
G0CYW2;embl-cds:AEG82505	Chaperone Protein Dnak	6	3	5	21	10	13
G0CYW6;embl-cds:AEG82509	Putative Membrane Protein				1	1	1

G0CYY1;embl-cds:AEG82523	Urease Accessory Protein Ureg	6	6	6	7	6	6
G0CYY2;embl-cds:AEG82524	Urease Accessory Protein Uref				1	1	1
G0CYY3;embl-cds:AEG82525	Urease Accessory Protein Uree	5	3	2	8	6	3
G0CYY4;embl-cds:AEG82526	Urease Subunit Alpha	5	4	4	7	5	5
G0CYY5;embl-cds:AEG82527	Urease Subunit Beta	4	2	3	6	3	3
G0CZ22;embl-cds:AEG81380	2-Oxoglutarate Dehydrogenase E1 Component	6	4	5	23	14	16
G0CZ24;embl-cds:AEG81382	Uncharacterized Protein				1	1	1
G0CZ35;embl-cds:AEG81393	Atp-Dependent Rna Helicase Dead	1		1	8	4	6
G0CZ52;embl-cds:AEG81410	Uncharacterized Protein	5	2	6	13	10	12
G0CZ53;embl-cds:AEG81411	Uncharacterized Protein	3	1	2	4	4	4
G0CZ57;embl-cds:AEG81415	Homoserine Dehydrogenase				5	2	4
G0CZ62;embl-cds:AEG81420	Peptide Chain Release Factor 1	2			2	4	4
G0CZ70;embl-cds:AEG81428	Atp Synthase Subunit Delta	4	2	3	11	7	6
G0CZ71;embl-cds:AEG81429	Atp Synthase Subunit Alpha	2	1	5	10	7	6
G0CZ73;embl-cds:AEG81431	Atp Synthase Subunit Beta	3	3	6	15	10	12
G0CZ86;embl-cds:AEG81444	Electron Transfer Flavoprotein Beta Subunit	3	2	2	5	3	3
G0CZ94;embl-cds:AEG82552	Phosphoenolpyruvate Carboxykinase [Gtp]	3	2	1	10	1	3
G0CZA6;embl-cds:AEG82564	Putative Secreted Protein	2	1	2	2	2	1
G0CZA9;embl-cds:AEG82567	Trehalose Corynomycolyl Transferase	6	5	6	5	5	5
G0CZB2;embl-cds:AEG82570	Uncharacterized Protein				2	1	1
G0CZB9;embl-cds:AEG82576	Udp-Galactopyranose Mutase	1	1	1		1	3
G0CZC6;embl-cds:AEG82583	Serine--Trna Ligase	6	2	5	7	7	7
G0CZC8;embl-cds:AEG82585	Putative Secreted Protein	1		1	2	1	1
G0CZF7;embl-cds:AEG82610	Uncharacterized Protein	1	1	1	4	2	1
G0CZG8;embl-cds:AEG82621	Uncharacterized Protein	5	2	3	4	4	3

G0CZH7;embl-cds:AEG81453	Aspartyl/Glutamyl-Trna(Asn/Gln) Amidotransferase Subunit C	3	1	1	4	3	4
G0CZI7;embl-cds:AEG81463	Aspartyl/Glutamyl-Trna(Asn/Gln) Amidotransferase Subunit B	6	4	5	11	6	7
G0CZP6;embl-cds:AEG81523	30S Ribosomal Protein S1	7	8	13	30	18	22
G0CZQ1;embl-cds:AEG81528	Uncharacterized Protein	2	1	2	3	2	2
G0CZQ5;embl-cds:AEG82627	Rod-Shaped Morphology Protein	4	3	2	8	8	10
G0CZQ7;embl-cds:AEG82629	Uncharacterized Protein	1	1		2	1	1
G0CZR3;embl-cds:AEG82636	50S Ribosomal Protein L9	3	3	4	6	4	5
G0CZR5;embl-cds:AEG82638	30S Ribosomal Protein S6			2	2	1	1
G0CZS0;embl-cds:AEG82643	Marr-Family Transcription Regulator	2		1	3	2	2
G0CZU4;embl-cds:AEG82666	N-Acylglucosamine-6-Phosphate 2-Epimerase				5	1	3
G0CZU9;embl-cds:AEG82671	Putative Membrane Protein				5	2	2
G0CTB4	Phenylalanine--Trna Ligase Beta Subunit				8	5	6
G0CTC5	Uncharacterized Protein				2	2	4
G0CTD0	Uncharacterized Protein				3	1	1
G0CTD1	Putative Secreted Protein				10	8	10
G0CTE1	Pseudouridine Synthase				1	1	2
G0CTE6	Putative Membrane Protein				3	1	3
G0CTE8	Iron Abc Transport System Substrate-Binding Protein				1	1	3
G0CTG2	Putative Secreted Protein				5	2	2
G0CTH4	Nadh Dehydrogenase				5	6	6
G0CTI0	Putative Secreted Protein				1	1	4
G0CTI6	Cobaltochelatase				2	2	2
G0CTJ0	Chromosome Partitioning Protein				5	4	4
G0CTJ8	Precorrin-8X Methylmutase				1	2	1
G0CTL9	Formate--Tetrahydrofolate Ligase				4	4	2
G0CTM0	Aspartate Ammonia-Lyase				7	4	5
G0CTM1	Putative Membrane Protein				1	2	2
G0CTN0	Putative Secreted Protein				8	6	6
G0CTP7	Uncharacterized Protein				1	1	1
G0CTQ1	Resuscitation-Promoting Factor Interacting Protein				7	5	4
G0CTQ3	Aconitase				15	9	8

G0CTR6	Putative Phage Tail Fiber Protein				4	2	5
G0CTW6	Transketolase				11	8	11
G0CTW7	Transaldolase				9	6	5
G0CTW8	Glucose-6-Phosphate 1-Dehydrogenase				5	1	1
G0CTW9	Uncharacterized Protein				4	1	2
G0CTX0	6-Phosphogluconolactonase				2	1	2
G0CTX3	Triosephosphate Isomerase				6	7	6
G0CTX4	Phosphoglycerate Kinase				14	9	10
G0CTX5	Glyceraldehyde-3-Phosphate Dehydrogenase				6	4	5
G0CTY2	Riboflavin Biosynthesis Protein Ribba				3	1	2
G0CTY6	Uncharacterized Protein				3	2	1
G0CTY7	Methionyl-Trna Formyltransferase				2	1	1
G0CTZ4	Guanylate Kinase				5	1	4
G0CTZ6	Orotidine 5'-Phosphate Decarboxylase				1	1	1
G0CTZ7	Carbamoyl-Phosphate Synthase Large Chain				12	8	6
G0CTZ8	Carbamoyl-Phosphate Synthase Small Chain				3	2	1
G0CU01	Bifunctional Protein Pyrr				2	1	3
G0CU05	N Utilization Substance Protein B Homolog				1	3	2
G0CU16	Dna Polymerase Iii Subunit Beta				2	3	3
G0CU26	Alanine--Trna Ligase				5	2	5
G0CU29	Aspartyl-Trna Synthetase				6	3	4
G0CU32	Putative Membrane Protein				5	2	5
G0CU36	L-Serine Dehydratase				5	2	3
G0CU37	Histidine--Trna Ligase				8	4	4
G0CU39	Probable Thiol Peroxidase				2	2	3
G0CU40	Peptidyl-Prolyl Cis-Trans Isomerase				5	1	1
G0CU41	Putative Secreted Protein				4	2	2
G0CU46	Protein Translocase Subunit Secd				5	2	1
G0CU63	Putative Secreted Protein				5	3	3
G0CU69	Uncharacterized Protein				2	3	3
G0CU81	Rna Polymerase Sigma Factor Siga				6	6	9
G0CU83	Uncharacterized Protein				1	2	2
G0CU88	Rna Polymerase Sigma Factor				3	3	4
G0CU90	Udp-Glucose 4-Epimerase				4	2	2
G0CUA8	Uncharacterized Protein				6	5	4
G0CUA9	Peptidyl-Prolyl Cis-Trans Isomerase				7	3	4
G0CUD3	Iron-Siderophore Binding Protein				3	6	8
G0CUH4	Uncharacterized Protein				1	2	2
G0CUI2	Putative Secreted Protein				5	5	5
G0CUI4	Phosphoenolpyruvate-Protein Phosphotransferase				8	5	5

G0CUJ9	Uncharacterized Protein				10	4	6
G0CUK2	Trna-2-Methylthio-N(6)-Dimethylallyladenosine Synthase				1	2	1
G0CUL5	Cell Division Protein				1	2	1
G0CUL7	Uncharacterized Protein				2	6	7
G0CUN5	Putative Secreted Protein				4	3	4
G0CUN6	Proline--Trna Ligase				5	3	3
G0CUP5	Uncharacterized Protein				3	1	2
G0CUP6	Probable Malate:Quinone Oxidoreductase				9	2	4
G0CUQ3	Penicillin-Binding Protein				7	7	7
G0CUR9	1,4-Alpha-Glucan-Branching Enzyme				1	1	5
G0CUS0	Putative Secreted Protein				8	6	7
G0CUT4	Crispr-Associated Protein				4	3	2
G0CUT7	Uncharacterized Protein				3	3	3
G0CUU0	Uncharacterized Iron-Regulated Membrane Protein				7	7	5
G0CUU1	Uncharacterized Protein				2	1	1
G0CUW2	Glycerol Kinase				6	1	2
G0CUW6	Uncharacterized Protein				1	1	1
G0CUW9	Arabinosyl Transferase				4	3	5
G0CUX2	Decaprenylphosphoryl-Beta-D-Ribose 2-Epimerase Component				10	4	7
G0CUY8	Putative Phenylalanine Aminotransferase				2	1	1
G0CUZ3	Uncharacterized Protein				4	5	4
G0CUZ9	Putative Secreted Protein				3	3	3
G0CV13	Uncharacterized Protein				8	4	2
G0CV19	30S Ribosomal Protein S16				6	3	3
G0CV20	Signal Recognition Particle Protein				8	5	7
G0CV23	Signal Recognition Particle Receptor Ftsy				3	3	4
G0CV32	Uncharacterized Protein				1	1	1
G0CV35	Uncharacterized Protein				1	1	1
G0CV40	Alpha-1,4 Glucan Phosphorylase				4	5	5
G0CV42	Pyruvate Kinase				8	6	6
G0CV45	Putative Membrane Protein				1	1	1
G0CV60	Glycogen Operon Protein Glgx Homolog				1	1	1
G0CV77	Isoleucine--Trna Ligase				15	7	13
G0CV91	Drug Exporter Of The Rnd Superfamily				2	1	1
G0CVA4	Uncharacterized Protein				1	2	3
G0CVA5	Uncharacterized Protein				3	2	3
G0CVC1	Putative Secreted Lpxtg Protein				5	5	4
G0CVC2	Putative Secreted Protein				6	6	6
G0CVD2	Uncharacterized Protein				1	4	7
G0CVD4	Laminin Subunit Beta-2				8	7	8
G0CVF2	Putative Secreted Protein				4	5	3
G0CVF4	Putative Secreted Protein				5	10	12

G0CVG7	Cell Division Protein				1	1	1
G0CVG8	Udp-N-Acetylmuramate--L-Alanine Ligase				5	3	4
G0CVG9	Udp-N-Acetylglucosamine--N-Acetylmuramyl-(Pentapeptide) Pyrophosphoryl-Undecaprenol N-Acetylglucosamine Transferase				1	2	3
G0CVH4	Udp-N-Acetylmuramyl-Tripeptide Synthetase				6	2	4
G0CVH5	Penicillin-Binding Protein				11	8	9
G0CVH6	Uncharacterized Protein				1	1	1
G0CVI4	Geranylgeranyl Pyrophosphate Synthase				7	3	3
G0CVI7	Serine/Threonine Protein Kinase				4	1	1
G0CVI8	Phospho-2-Dehydro-3-Deoxyheptonate Aldolase				4	3	6
G0CVJ4	Cell Wall-Associated Hydrolase				3	3	2
G0CVK2	Asparagine Synthetase				10	2	7
G0CVK4	Uncharacterized Protein				3	1	1
G0CVK9	Probable Cytosol Aminopeptidase				8	1	1
G0CVL1	Dihydrolipoamide Acyltransferase				16	5	5
G0CVL2	Glycine Dehydrogenase (Decarboxylating)				4	1	1
G0CVL3	Aminomethyltransferase				5	5	4
G0CVL4	Glycine Cleavage System H Protein				2	1	1
G0CVM5	Glutamine Synthetase				1	1	2
G0CVN4	Threonine Synthase				6	6	2
G0CVN8	Heme Oxygenase				1	3	2
G0CVP2	Uncharacterized Protein				5	1	2
G0CVR3	Dna Topoisomerase 1				9	1	4
G0CVS7	Putative Secreted Protein				11	7	9
G0CVU6	Uncharacterized Protein				15	9	4
G0CVV7	Putative Secreted Protein				6	5	5
G0CVX2	Uncharacterized Protein				5	4	2
G0CVZ0	Pyruvate Dehydrogenase E1 Component				19	6	7
G0CW01	Putative Secreted Protein				2	1	1
G0CW05	Putative Secreted Lpxtg Protein				3	1	1
G0CW08	Glycine--Trna Ligase				7	4	5
G0CW14	Gtpase Era				2	1	5
G0CW15	Pyridoxamine Kinase				2	1	1
G0CW20	Chaperone Protein Dnaj				3	3	4
G0CW28	Peptidyl-Dipeptidase				4	2	1
G0CW33	Putative Secreted Protein				3	2	2
G0CW41	Putative Secreted Protein				2	7	8
G0CW72	2-Succinyl-5-Enolpyruvyl-6-Hydroxy-3-Cyclohexene-1-Carboxylate Synthase				1	1	1
G0CW75	Demethylmenaquinone Methyltransferase				1	1	2

G0CW80	Transcription Termination/Antitermination Protein Nusg				4	2	1
G0CW83	Bleomycin Hydrolase				2	1	1
G0CW85	Uncharacterized Protein				1	1	1
G0CW93	Putative Secreted Protein				5	1	1
G0CW94	Putative Secreted Protein				3	7	3
G0CW96	Dna-Directed Rna Polymerase				23	14	13
G0CW98	Putative Secreted Lpxtg Protein				4	3	3
G0CWD5	Uncharacterized Protein				1	1	3
G0CWE4	Non-Specific Acid Phosphatase				9	5	5
G0CWE6	50S Ribosomal Protein L6				6	3	4
G0CWF3	Uncharacterized Protein				1	1	1
G0CWH0	Putative Secreted Protein				4	3	2
G0CWH1	Uncharacterized Protein				3	1	1
G0CWJ2	Uncharacterized Protein				1	1	1
G0CWK1	Uncharacterized Protein				1	2	1
G0CWK7	Ribosomal Silencing Factor Rsfs				1	2	2
G0CWL7	Ribonuclease E				10	5	14
G0CWM2	Dihydrofolate Synthase / Folylpolyglutamate Synthase				5	3	1
G0CWM3	Valine--Trna Ligase				13	4	6
G0CWN1	Trigger Factor				9	6	7
G0CWP1	Uncharacterized Protein				4	2	1
G0CWP3	Adenylate Kinase				6	5	6
G0CWP5	Sialidase				20	14	17
G0CWP6	Putative Secreted Protein				4	2	3
G0CWS8	60 Kda Chaperonin				18	12	11
G0CWT4	Inosine-5'-Monophosphate Dehydrogenase				4	3	3
G0CWT8	Gmp Synthase [Glutamine-Hydrolyzing]				11	4	6
G0CWU7	Putative Secreted Protein				1	1	1
G0CWV2	Manganese Abc Transporter, Atp-Binding Protein				1	1	1
G0CWX8	Uncharacterized Protein				13	7	5
G0CWZ3	Putative Secreted Protein				5	1	4
G0CWZ4	Putative Secreted Protein				5	4	4
G0CWZ6	Corynebacterineae Mycolate Reductase A				2	2	1
G0CX25	Fatty Acid Synthase				47	25	30
G0CX30	Uncharacterized Protein				10	5	5
G0CX36	Ribonuclease Ph				2	2	1
G0CX49	Isocitrate Dehydrogenase				17	13	17
G0CX54	Venom Serine Protease Kn13				1	1	1
G0CX59	Uncharacterized Protein				6	3	4
G0CX68	Pyruvate Carboxylase				26	12	11
G0CX70	Uncharacterized Protein				1	1	1
G0CX71	Acyl-Coa Carboxylase Alpha Subunit				19	12	11
G0CX73	Uncharacterized Protein				7	4	5
G0CX77	Ribokinase				1	2	2

G0CX80	Acyl-Coa Carboxylase Complex Subunit				6	8	4
G0CX95	Putative Secreted Protein				7	12	10
G0CX98	Putative Glycosyltransferase				2	2	1
G0CXA3	Phosphomannomutase				5	4	4
G0CXA8	Adenosylhomocysteinase				2	1	1
G0CXB2	Lipoprotein Lpqb				4	3	3
G0CXB4	Uncharacterized Protein				5	3	3
G0CXB5	Protein Translocase Subunit Seca				15	10	14
G0CXC0	Uncharacterized Protein				6	5	5
G0CXD0	Putative Secreted Protein				1	3	3
G0CXE4	Uncharacterized Protein				4	3	3
G0CXE5	Putative Secreted Protein				8	3	3
G0CXE8	Putative Secreted Protein				1	1	2
G0CXF4	Uncharacterized Protein				1	1	1
G0CXF9	Cysteine Synthase				10	4	3
G0CXG8	Trna-Dihydrouridine Synthase				2	1	1
G0CXI2	Phosphoribosylformylglycinamidine Cyclo-Ligase				1	1	2
G0CXI8	Phosphoribosylformylglycinamidine Synthase Subunit Purl				3	4	4
G0CXJ4	Adenylosuccinate Lyase				7	4	4
G0CXJ7	Phosphoribosylamine--Glycine Ligase				6	7	6
G0CXK4	Putative Secreted Protein				10	10	9
G0CXL8	Uncharacterized Protein				5	4	5
G0CXL9	Putative Secreted Protein				1	2	2
G0CXM2	Upf0182 Protein Culc22_00609				9	10	7
G0CXN6	Putative Secreted Protein				5	5	4
G0CXN9	Peptide Chain Release Factor 2				4	2	3
G0CXP7	Putative Iron Abc Transport System, Solute-Binding Protein				11	6	5
G0CXR4	Uncharacterized Protein				1	2	3
G0CXS5	Putative Secreted Protein				1	1	1
G0CXU0	Putative Secreted Lpxtg Protein				3	1	1
G0CXU1	Uncharacterized Protein				3	3	3
G0CXU9	Putative Adhesin				7	6	7
G0CXY6	Uncharacterized Protein				4	1	1
G0CXZ7	Uncharacterized Protein				1	1	1
G0CY02	Cysteine--Trna Ligase				4	1	1
G0CY07	Putative Secreted Protein				2	2	1
G0CY14	Atp-Dependent Clp Protease Atp-Binding Subunit				13	5	5
G0CY18	Uncharacterized Protein				1	1	1
G0CY21	Laminin Subunit Beta-4				13	12	12
G0CY38	Glucose-6-Phosphate Isomerase				3	3	2
G0CY45	Bifunctional Purine Biosynthesis Protein Purh				9	3	3
G0CY47	Glutamate Abc Transport System Substrate-Binding Protein				3	4	3
G0CY52	Uncharacterized Protein				1	1	2

G0CY61	Trypsin-Like Serine Protease				12	4	7
G0CY77	Methionine--Trna Ligase				1	2	4
G0CY79	Resuscitation-Promoting Factor				3	1	1
G0CY90	Uncharacterized Protein				1	2	1
G0CY93	Glyceraldehyde-3-Phosphate Dehydrogenase				11	6	8
G0CY98	Uncharacterized Protein				1	2	1
G0CYA8	Lysine--Trna Ligase				6	9	7
G0CYB2	Betaine Aldehyde Dehydrogenase				1	2	2
G0CYB7	Uncharacterized Protein				1	1	2
G0CYC6	D-Alanyl-D-Alanine Carboxypeptidase				7	6	6
G0CYC8	Uncharacterized Protein				2	1	2
G0CYD0	Putative Secreted Protein				2	4	1
G0CYD3	Uncharacterized Protein				5	3	3
G0CYD5	Uncharacterized Protein				5	3	3
G0CYD6	Uncharacterized Protein				4	1	2
G0CYD7	Uncharacterized Protein				1	1	1
G0CYD8	Uncharacterized Protein				9	5	6
G0CYE0	60 Kda Chaperonin				16	12	9
G0CYE1	Uncharacterized Protein				4	1	1
G0CYF3	Putative Secreted Protein				2	1	1
G0CYG5	Acetate Kinase				12	6	5
G0CYG6	Phosphate Acetyltransferase				10	5	6
G0CYG7	Ferredoxin/Ferredoxin-Nadp Reductase				14	12	8
G0CYH6	Uncharacterized Protein				1	1	1
G0CYH7	Uncharacterized Protein				5	2	4
G0CYH8	Uncharacterized Protein				4	3	4
G0CYI9	Putative Secreted Protein				8	8	9
G0CYJ2	Bifunctional Protein Glmu				12	4	6
G0CYL6	Serine Hydroxymethyltransferase				7	2	3
G0CYM0	Putative Secreted Protein				4	1	2
G0CYM5	Fructose-1,6-Bisphosphatase				5	3	2
G0CYM9	4-Hydroxy-3-Methylbut-2-Enyl Diphosphate Reductase				2	2	2
G0CYN1	Putative Membrane Protein				1	1	1
G0CYN2	Ribosome-Binding Atpase Ychf				7	4	4
G0CYP6	Putative Secreted Protein				4	8	8
G0CYQ2	Macrolide Export Atp-Binding/Permease Protein				3	2	3
G0CYQ4	Uncharacterized Protein				2	2	1
G0CYQ6	Putative Secreted Protein				6	3	3
G0CYQ8	Uncharacterized Protein				1	1	2
G0CYR6	2,3,4,5-Tetrahydropyridine-2,6-Dicarboxylate N-Succinyltransferase				4	3	3
G0CYS6	Putative Secreted Lpxtg Protein				19	13	12
G0CYS7	Putative Secreted Protein				67	37	38
G0CYT4	Glycerol-3-Phosphate Dehydrogenase				7	9	9

G0CYU2	Uncharacterized Protein				3	1	1
G0CYU4	Laminin Subunit Alpha-2				2	5	6
G0CYU5	Putative Secreted Protein				6	4	4
G0CYU9	Uncharacterized Protein				3	1	2
G0CYV0	Putative Membrane Protein				6	2	3
G0CYV2	Uncharacterized Protein				20	11	12
G0CYW1	Protein Grpe				4	4	1
G0CYW3	Surface-Anchored Protein, Fimbrial Subunit				6	5	4
G0CYX8	Abc Transport System, Substrate-Binding Protein				9	4	4
G0CYY7	Putative Membrane Protein				2	1	1
G0CYY8	Aspartate Aminotransferase				3	1	2
G0CYZ0	Lysyl-Trna Synthetase				4	2	3
G0CZ02	Uncharacterized Protein				17	11	9
G0CZ04	Arabinofuranosyl Transferase D				1	2	3
G0CZ09	Ribosomal Rna Methyltransferase				7	4	4
G0CZ10	Uncharacterized Protein				2	1	2
G0CZ21	Putative Secreted Protein				1	2	3
G0CZ34	Galactokinase				1	3	2
G0CZ55	Arginine--Trna Ligase				12	5	10
G0CZ61	Transcription Termination Factor Rho				13	11	9
G0CZ64	Uncharacterized Protein				1	1	1
G0CZ69	Atp Synthase Subunit B				6	6	6
G0CZ72	Atp Synthase Gamma Chain				2	3	4
G0CZ79	Uncharacterized Protein				3	3	2
G0CZ80	Uncharacterized Protein				1	1	1
G0CZ87	Electron Transfer Flavoprotein Alpha Subunit				2	3	2
G0CZA3	Polyketide Synthase				12	5	7
G0CZA4	Acyl-Coa Synthetase				15	14	16
G0CZA5	Envelope Lipids Regulation Factor				4	3	2
G0CZA7	Trehalose Corynomycolyl Transferase				22	7	6
G0CZB3	Glycosyltransferase				7	3	2
G0CZB7	Glycerophosphoryl Diester Phosphodiesterase				5	11	13
G0CZC3	Putative Secreted Protein				10	10	10
G0CZD6	Cell-Surface Hemin Receptor				6	5	2
G0CZD9	Uncharacterized Protein				3	2	4
G0CZE3	Thymidine Phosphorylase				1	2	2
G0CZE8	Superoxide Dismutase				1	1	2
G0CZE9	Uncharacterized Protein				1	1	1
G0CZG3	Housekeeping Sortase				5	3	2
G0CZH8	Glutamyl-Trna(Gln) Amidotransferase Subunit A				11	3	5
G0CZJ9	Ketol-Acid Reductoisomerase				5	4	3
G0CZK0	Putative Membrane-Anchored Protein				6	3	2

G0CZK4	D-3-Phosphoglycerate Dehydrogenase				6	5	7
G0CZL2	Glutamate--Trna Ligase				9	7	5
G0CZL4	Putative Secreted Protein				14	8	10
G0CZM9	D-Alanine--D-Alanine Ligase				4	3	3
G0CZP4	Dna Polymerase				2	1	2
G0CZP7	Pts System Glucose-Specific Component Ii				4	2	1
G0CZQ3	Uncharacterized Protein				4	4	2
G0CZQ9	Putative Secreted Protein				4	1	1
G0CZR8	Penicillin-Binding Protein				15	9	12
G0CZS4	Upf0176 Protein Culc22_02279				2	1	1
G0CZS6	Cell-Surface Hemin Receptor				4	3	4
G0CZT1	Putative Membrane Protein				12	8	8
G0CZT2	Leucine--Trna Ligase				13	2	9
G0CZU2	Glucosamine-6-Phosphate Deaminase				4	1	2
G0CZV7	Uncharacterized Protein				3	4	3
G0CZY6	Putative Secreted Protein				10	1	2
G0CZY7	Putative Membrane Protein				3	2	4
